# Oxidative Dissolution Effects on Shale Pore Structure, Mechanical Properties, and Gel-Breaking Performance

**DOI:** 10.3390/gels11120982

**Published:** 2025-12-07

**Authors:** Jingyang Chen, Liangbin Dou, Tao Li, Yanjun Zhang, Kelong Deng, Xuebin Cheng, Zhifa Kang, Ruxu Wang, Yang Shi

**Affiliations:** 1 College of Petroleum Engineering, Xi’an Shiyou University, Xi’an 710065, China; 24111010029@stumail.xsyu.edu.cn (J.C.);; 2 Engineering Research Center for Development and Management of Western Low-Permeability and Ultra-Low Permeability Oilfield, Ministry of Education, Xi’an Shiyou University, Xi’an 710065, China; 3 Natural Gas Research Institute of Shaanxi Yanchang Petroleum (Group) Co., Ltd., Xi’an 710065, China

**Keywords:** shale gas reservoir, oxidative dissolution, pore structure evolution, wettability alteration, compressive strength, elastic modulus, gel-breaking performance

## Abstract

Shale reservoirs contain abundant organic matter, pyrite, and clay minerals, making them highly susceptible to fluid-sensitivity damage; consequently, conventional hydraulic fracturing often yields poor stimulation performance, with low fracturing fluid flowback and rapid post-treatment production decline. Oxidative dissolution, however, can significantly alter the physical properties of shale reservoirs and improve stimulation effectiveness. In this study, nuclear magnetic resonance (NMR), contact-angle measurements, and triaxial compression tests are combined to systematically evaluate the effects of oxidative dissolution on the pore structure, wettability, and mechanical properties of Wufeng Formation shale from the Sichuan Basin. Core-flooding experiments with NaClO solutions show that, as the oxidant dosage (pore volume) increases, shale permeability rises by 66.67–266.67% and porosity by 1.79–9.58%, while the hydrophilic surface fraction increases from 5.45% to 61.73%. These changes are accompanied by a steady reduction in rock strength: the compressive strength decreases by up to 57.8%, and the elastic modulus exhibits a non-monotonic response to oxidation. Oxidative dissolution preferentially enlarges micropores, improves pore connectivity, and strengthens water wetness by consuming oil-wet organic matter and pyrite, which also enhances gel-breaking efficiency. Based on the experimental results, a series of characterization models are developed for oxidized shale reservoirs, including quantitative relationships linking porosity to compressive strength, elastic modulus, and contact angle, as well as a model relating oxidant dosage to microscopic pore structure evolution and imbibition enhancement. Overall, the coupled modifications of pore structure, wettability, and mechanical behavior produced by oxidative dissolution synergistically broaden the effective action range of fracturing fluids, promote shale gas desorption, and improve hydrocarbon seepage, providing a theoretical basis and practical guidance for oxidation-assisted stimulation in shale reservoirs.

## 1. Introduction

Shale reservoirs, which contain extensive amounts of organic matter, pyrite, and various minerals, are characterized by ultra-low porosity and permeability, a multi-scale distribution of pores, a variety of micro-cracks, and a complex spatial structure [1]. Hydraulic fracturing is the predominant technology for stimulating and transforming shale reservoirs; however, its overall development efficacy is suboptimal due to the rapid decline in production observed following fracturing. It has been found that the primary recovery rate following hydraulic fracturing is low, with the flowback rate of the fracturing fluid generally being less than 40% [2]. Furthermore, the high content of clay minerals in the shale reservoir can result in serious formation damage and limit the capabilities of reservoir reconstruction [3]. Employing chemical methods to oxidize and dissolve organic matter, pyrite, and other components in shales can effectively increase the reservoir porosity and improve shale gas mobility. Additionally, the synergistic effects of various reconstruction factors, such as oxidation, dissolution, acidification, and dissolution self-supporting effect, can effectively improve the reservoir physical properties, providing a theoretical and methodological foundation for the application of the oxidation and dissolution techniques in the development of shale oil and gas resources [4]. This study introduces a novel approach for transforming shale reservoirs that holds considerable value for enhancing resource development of shale resources.

On a broader scale, the evolution of unconventional hydrocarbon exploitation has been accompanied by escalating concerns over stimulation efficiency and environmental footprint. Recent reviews, such as that by Kokkinos et al. (2022) [5], have emphasized that the successful development of shale, tight sandstone, and other unconventional reservoirs demands stimulation technologies that not only boost hydrocarbon recovery but also minimize chemical inputs and formation damage across the reservoir life cycle. In this context, oxidative dissolution emerges as a promising stimulation concept, as it couples targeted modification of rock microstructure with the potential to integrate oxidants into established hydraulic fracturing workflows (e.g., oxidative gel-breaking systems) [5].

Beyond these bulk-scale considerations, investigations of the Wufeng–Longmaxi shale have further revealed that pore–fracture connectivity is governed more by lamina-scale heterogeneity than by bulk petrophysical properties alone. Complementing this macroscopic perspective, Xie et al. (2025) [6] systematically probed the impact of laminae characteristics on pore–fracture connectivity in the Wufeng–Longmaxi shale through the integration of thin-section observations, SEM imaging, and permeability anisotropy measurements. They demonstrated that the thickness, composition, and stacking patterns of clay-rich and silty laminae dictate the connectivity between matrix pores and fractures, thereby inducing pronounced horizontal–vertical permeability anisotropy and strongly shaping gas transport pathways in this interval. These findings indicate that lamina-controlled pore–fracture systems constitute the primary migration pathways for shale gas, yet their connectivity is readily compromised by diagenetic cementation and mechanical compaction. Leveraging this lamina-scale insight, the present study focuses on how oxidative dissolution modifies pore networks and pore-mechanical coupling in Wufeng shale cores, and whether chemical stimulation can effectively reopen or enhance lamina-governed pore–fracture connectivity [6].

By comparing the removal degree of organic matter from shales in different blocks under the action of an oxidant, Kuila et al. (2014) found that the mineral components and organic matter content in shales can significantly influence the effectiveness of oxidative dissolution, thereby demonstrating the feasibility of oxidative dissolution [7,8]. Cheng et al. (2021) [9] studied the oxidative dissolution of Longmaxi organic-rich shale using H_2_O_2_, combining kinetic tests on crushed/sliced samples (2–10 wt%, 40–80 °C) with stress-sensitivity tests on fractured plugs; they quantified OM removal and Arrhenius-type rates and showed that oxidation widens fractures and lowers stress sensitivity, suggesting H_2_O_2_ can help maintain unpropped fracture conductivity [9]. Additionally, it is found that the acidity of the solution increases after such a reaction, which further indicates that the oxidative dissolution of shale is accompanied not only by the oxidation reaction but also by acid etching. This demonstrates that the oxidant solution can effectively improve the porosity and permeability of shale reservoirs through this combined mechanism [10]. Donmoyer S et al. (2022) [11] conducted batch reaction experiments using Marcellus shale and common oxidants (persulfate, bromate, hypochlorite) to investigate oxidative dissolution. Geochemical and mineralogical analyses revealed that these oxidants induce pyrite/organic matter oxidation and carbonate dissolution, accompanied by scale formation and increased dissolved inorganic carbon (DIC)/sulfate concentrations. Notably, bromate and hypochlorite leach more trace elements and halogenated volatile organic compounds (VOCs) than persulfate [11].

Beyond batch oxidative dissolution tests, recent studies have refined the mechanistic understanding of persulfate-based oxidants that are widely used both as fracturing fluid additives and as gel breakers. Transition metal-activated or thermally activated persulfate systems generate sulfate and hydroxyl radicals that attack organic polymers and organic matter in the rock matrix via radical chain reactions, with reaction pathways and efficiencies strongly controlled by temperature, pH, and the presence of carbonate or clay buffers. For organic-rich shales, kinetic experiments have shown that oxidants such as H_2_O_2_ and persulfate follow Arrhenius-type relationships, with activation energies that depend on organic matter type and mineralogy, and that oxidation can significantly widen existing fractures and reduce stress sensitivity. These insights provide a mechanistic foundation for the oxidant–rock interactions investigated in this study [12].

To explore the influence of organic matter and other minerals on shale wettability, Cheng et al. (2020) [13] tested the shale wettability after oxidation dissolution. Their experimental results show that oxidation dissolution can effectively improve the wettability of shale surface and increase the pore radius, greatly shortening the self-absorption time of shale and significantly increasing the self-absorption capacity of the entire reservoir. Moreover, these findings suggest that oxidative dissolution in shale reservoirs can alleviate the water lock effect, improve the imbibition development effect, and promote the fracture expansion, thereby effectively improving the permeability of shale reservoirs [13]. In terms of the specific mechanism of oxidative dissolution, Alrubh J et al. (2025) [14] exposed Eagle Ford shale cores to 30% H_2_O_2_ (in the presence/absence of slickwater) at 120 °F for 2–4 h. Via SEM/EDX, TOC, gravimetric, and elastic measurements, they observed enhanced microporosity, organic matter degradation accompanied by slight mass loss, and a 30–60% reduction in Young’s modulus—confirming that H_2_O_2_-induced oxidative dissolution softens shale [14]. Beyond oxidant types, the regulatory role of shale’s own mineral composition in the oxidative dissolution process has also garnered attention. Ferguson et al. (2021) [15] isolated carbonate effects on shale oxidative dissolution by autoclaving carbonate-rich Marcellus shale and its decarbonated counterpart with synthetic fracturing fluid (100 °C, ~2500 psi, 14 days), monitoring pH, ions/metals, VOCs, DIC, SEM-EDS, and saturation indices. They found that carbonates buffer pH and scavenge oxidants, suppressing clay dissolution, pyrite oxidation, and metal release; in contrast, carbonate-free conditions maintain low pH and promote clay/pyrite dissolution. This confirms that carbonate content governs oxidation, scaling, and porosity evolution during shale–fluid interactions [15]. In addition to mineral dissolution and mechanical damage, water sorption by shale organic matter can markedly influence pore accessibility and transport properties. Fu et al. (2025) [16] found that water–organic matter interactions in bauxite reservoirs similarly regulate pore structure evolution. Recent research has shown that sorbed water can significantly modify the micropore system and thus affect gas storage and flow characteristics. These moisture-related effects are closely related to the oxidative treatments considered in this study, because our experiments are conducted under water-saturated conditions where sorbed water participates in both organic matter oxidation and mechanical weakening [16].

Numerous scholars have performed extensive research on the oxidation effect of commonly used oxidants. In their investigation into oxidative dissolution within Longmaxi Formation shales, Chen et al. (2017) [17] found that H_2_O_2_ effectively dissolves pyrite along with other minerals at room temperature, resulting in enhanced pore space due to the formation of dissolution voids. Zhou et al. (2018) [18] performed a comparative analysis of the oxidation and dissolution effects of H_2_O_2_ and sulfate-based oxidants. They observed that the mineral composition of the shale remained almost unchanged after H_2_O_2_ oxidation; however, the persulfate oxidation of shale core samples led to the subsequent production sediment as large quantities of carbonate minerals were dissolved. Those results indicate that acid oxidants primarily dissolve carbonate minerals in shales, with pyrite and organic matter undergoing partial oxidation reactions. In practice, it is essential to determine the mineral composition characteristics of shale and select appropriate oxidants to achieve the optimal oxidation dissolution effect [17,18]. Cheng et al. (2021) [19] compared the responses of various blocks to H_2_O_2_-induced oxidative dissolution, revealing distinct decomposition patterns for organic matter in regions characterized by either carbon–hydrogen bond fracture or carbon–oxygen bond fracture. Their findings demonstrated the differences in the development of organic matter within shale reservoirs can significantly affect the oxidative dissolution effects [19,20]. Yang et al. (2020) [21] evaluated the effects of H_2_O_2_, NaClO, and Na_2_S_2_O_8_ oxidants on reservoir dissolutions within Wufeng Formation shale. They found that acidic oxidants predominantly target carbonate minerals, whereas alkaline ones focus more on aluminosilicate components. Most oxidants will produce a large amount of gas at high temperature or during their interaction with shale, which can significantly supplement formation energy. In association with oxidation dissolution, such a phenomenon contributes to creating new fractures and extending them within the reservoir. According to the research results, it can be concluded that the oxidant NaClO primarily targets organic matter and pyrite in shale, effectively increasing the pore space and pore radius in the reservoir. However, the oxidant Na_2_S_2_O_8_ mainly decomposes carbonate minerals in shale during the acid dissolution process due to its strong acidity. Furthermore, it demonstrates that oxidative dissolution can change the wettability of shale by modifying the functional groups on its surface, thereby enhancing its spontaneous imbibition effect and improving the mobility of oil and gas [21,22]. Liang et al. (2021) [23] found that acid dissolution and oxidation are the two primary aspects of the dissolution effect of Na_2_S_2_O_8_ on Marcellus shale, with acid dissolution being dominant in the reaction. Specifically, the reaction effect of calcite and dolomite being dissolved by acid is more pronounced than that of pyrite in the oxidation reaction [23,24,25].

It is clear from existing studies that the oxidative dissolution effect of different oxidants on a range of reservoir types differs markedly. Most existing research focuses on the pore throat characteristics and action mechanisms of oxidative dissolution in shale, with limited attention paid to the mechanical properties of shale after oxidative dissolution [26,27]; further, research on the response relationship between shale porosity and mechanical properties post-oxidation is even more scarce [28,29,30]. Beyond these gaps in shale-focused research, prior research on oxidative acidizing in carbonates and carbonate-rich shales has mainly centered on carbonate dissolution, pH buffering, and scale formation during prolonged exposure to oxidizing fracturing fluids—without systematically quantifying the accompanying changes in rock mechanical behavior [15]. In parallel, a large body of research on oxidizing gel breakers (e.g., persulfate-, permanganate-, and hypochlorite-based breakers) has concentrated on polymer degradation kinetics and residue removal, typically treating the reservoir rock as chemically inert. Even other studies that have investigated shale dissolution in hydraulic fracturing fluid systems or emphasized mineral-selective dissolution and wettability alteration have seldom linked mineralogy-controlled oxidative dissolution to both pore structure evolution and mechanical degradation of shale cores or to the subsequent gel-breaking response of actual fracturing fluids.

In this study, by means of rock mechanics experiments and nuclear magnetic resonance (NMR), we compare the changes in pore and mechanical characteristics of shale reservoirs before and after oxidative dissolution, clarify the influence of oxidative dissolution on pore and mechanical properties as well as their response relationship, investigate various reaction mechanisms during oxidation dissolution, and accurately evaluate the treatment’s impacts on shale physical properties. Concurrently, addressing the gap that existing gel-breaker research treats reservoir rock as chemically inert, we quantify the oxidative gel-breaking efficiency under actual shale reservoir conditions. Based on the experimental results, we develop a dynamic model for oxidation-enhanced imbibition that evaluates the multi-field effects of oxidation–imbibition, mechanical damage, and gel-breaking behavior, providing a feasible tool to optimize the scope, mode, and control of oxidative fluid application during hydraulic fracturing. Ultimately, this study develops a new method and technology for shale gas exploitation under the coupled, mutually feedback-driven multi-field effects (oxidative dissolution, gel breaking, pore mechanics), offering technical guidance for shale reservoir development and filling a significant gap in integrating oxidative gel-breaking performance with rock property modifications.

To better position this study within the existing body of research on chemical stimulation and shale–fluid interactions, Table 1 summarizes several representative related studies and compares them with the present study in terms of target formation, fluid/oxidant system, main objectives, characterization methods, and key limitations. As shown in the last row, this study integrates pore structure evolution, wettability alteration, mechanical weakening, and oxidant gel-breaking performance for Wufeng Formation shale, thereby highlighting the specific novelties of our contribution relative to previous studies.

## 2. Results and Discussion

### 2.1. Effect of Oxidative Dissolution on Physical Properties of Shale Reservoir

**Figure 1:**
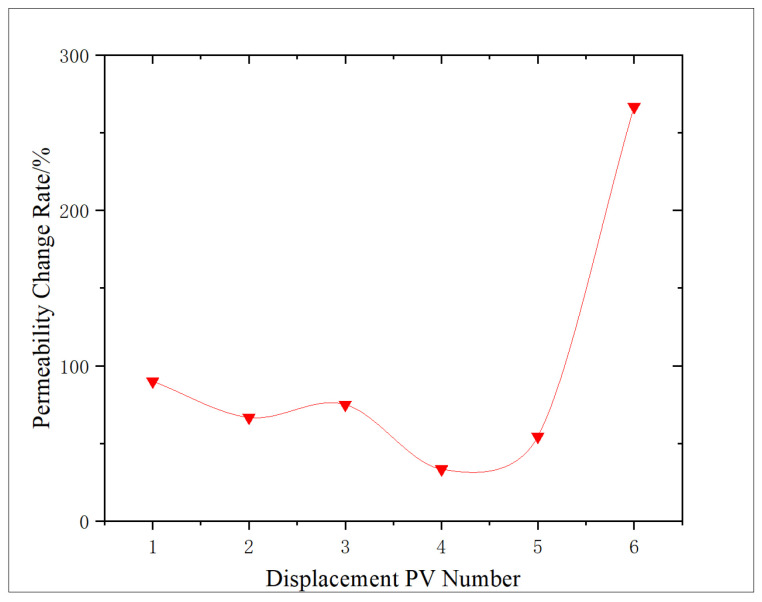
Variation in permeability rate with PV number.

**Figure 2:**
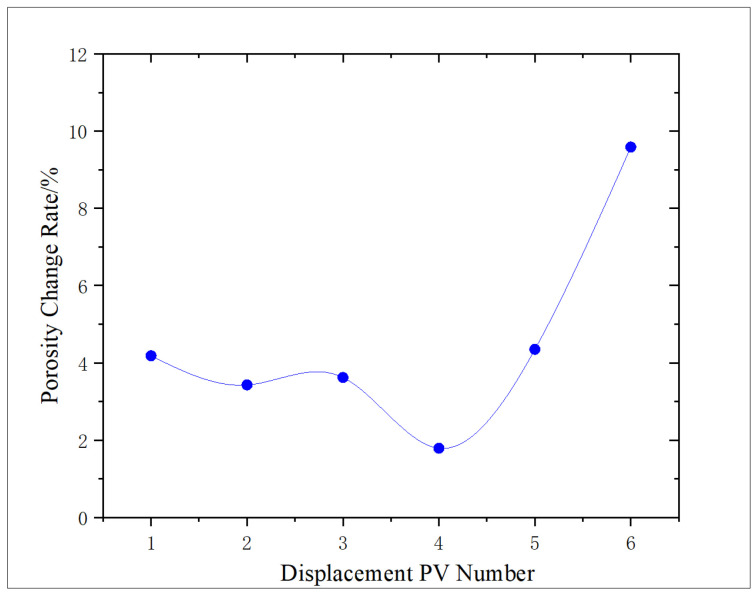
Variation in porosity rate with PV number.

The change characteristics of shale porosity and permeability under different amounts of NaClO oxidation solution were analyzed, and the effect of oxidation dissolution of oxidation solution was evaluated. The experimental results are shown in Figure 1 and Figure 2.

Through Figure 1, the permeability change characteristics of shale before and after oxidation dissolution are analyzed. Comparing the permeability of shale before oxidation dissolution, it can be seen that, after oxidation dissolution, the permeability of shale samples obviously increased, with an increase range of 66.7–266.7%. According to the result analysis curve, it can be seen that the increase in core permeability reaches 90.0% during 1 PV displacement. The analysis is that the oxidation solution enters the core and produces a large amount of gas at high temperature, which leads to some micro-cracks in the core, resulting in increased permeability. With the increase in the amount of oxidation solution, the increase in core permeability shows a downward trend. The main reason for the analysis is that, with the increase in the action amount and the extension of the action time, ferric hydroxide and other sediments may be produced, blocking part of the pores. Moreover, micro-fractures lead to the decrease in gas fracturing effect, and some gases may also lead to the Jamin effect, which causes the increase in permeability to decrease (to the lowest at 4 PV). However, with the continuous increase in the action amount (above 5 PV), some tiny sediments and stripped rock fragments can be carried out of the core, and some organic acids generated by organic matter reaction decompose the sediment, and concurrently, dissolution makes the shale pores and micro-crack surfaces irregular to produce a self-supporting effect, thus increasing the permeability of the core, even increasing the permeability by more than 200.0%.

According to Figure 2, the influence of oxidation dissolution on shale porosity is analyzed. Compared with the shale porosity before oxidation dissolution, it is concluded that the increase range of oxidation dissolution in shale porosity is 1.79–9.58%, and the overall change trend of porosity is similar to that of permeability, which shows a trend of decreasing first and then increasing with the increase in the amount of oxidation solution. This phenomenon is mainly due to the fact that, when the amount of oxidizing solution is low, some minerals such as pyrite, organic matter, and carbonate are dissolved by oxidants, resulting in a large number of dissolved pores, which increases the porosity of shale. However, with the increase in the amount of action, Fe^3+^ in the alkaline environment for a long time produces partial sediment, resulting in a relative decrease in the increase in porosity (reaching the lowest point at 4 PV). As the action amount continues to increase, the content of pyrite decreases, which reduces the sediment, and some organic acids produced by organic matter reaction decompose some sediment. Moreover, a large amount of oxidation solution carries the sediment out of the core, which leads to a much higher increase in porosity than that of low action amount.

As shown in Figure 1 and Figure 2, both the permeability and porosity change rates reach a minimum at approximately 4 PV of oxidizing solution displacement. At low PV, the oxidation of organic matter and pyrite, together with the generation of organic acids, leads to an overall increase in pore volume and permeability. However, prolonged reaction in an alkaline environment also produces secondary sediments and mobilized fines that partially accumulate in the pore network, resulting in a temporary reduction in the rates of permeability and porosity increase and even a relative decrease (minimum) at 4 PV. When the injected volume exceeds 4 PV, the additional oxidizing solution progressively dissolves and flushes out these reaction products, and the dissolution of the matrix becomes dominant again, so porosity and permeability resume increasing.

### 2.2. Effect of Oxidation Dissolution on Shale Wettability

**Figure 3:**
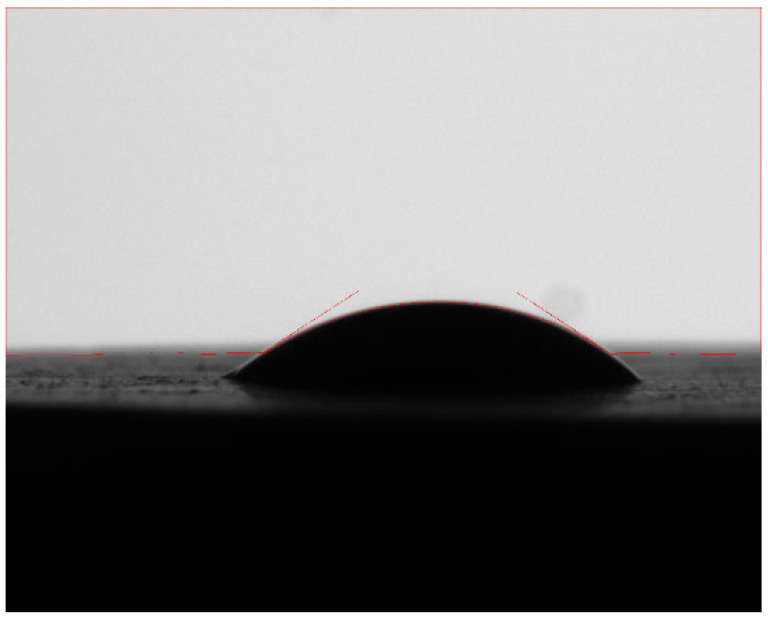
Wetting angle of No. 4 core before oxidative dissolution.

**Figure 4:**
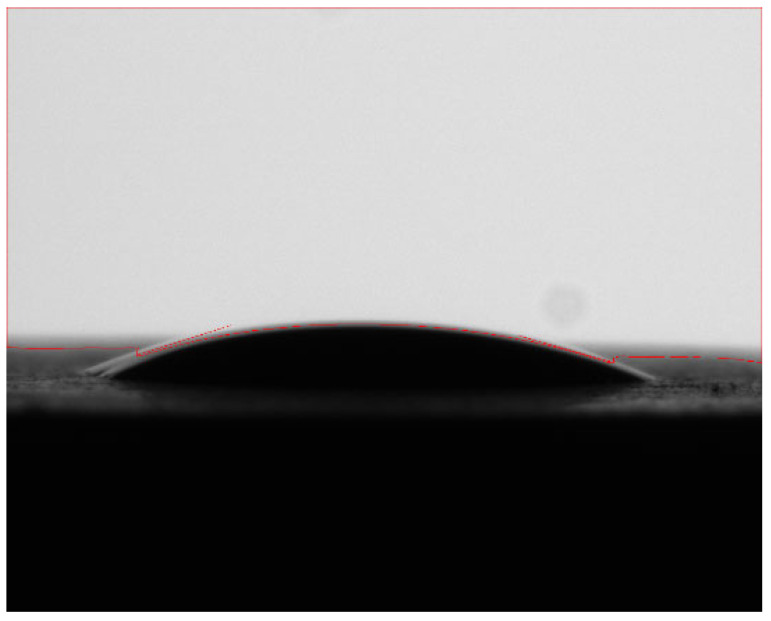
Wetting angle of No. 4 core after oxidation dissolution.

The wetting angle of shale samples before and after oxidation dissolution was measured by a contact-angle tester, and the experimental results are shown in Table 2. The wetting angle before oxidation dissolution ranges from 8.1 to 34.1 (Figure 3), and the wettability of the core in the study area is mainly hydrophilic. The wetting angle of shale is between 3.1 and 20.9 after oxidation dissolution (Figure 4), which shows that oxidative dissolution makes the hydrophilicity of shale samples stronger.

With the increase in the amount of oxidation solution, the hydrophilicity of shale increases, ranging from 5.45% to 61.73%. At 5 PV, an abnormally high value is observed. In association with the analysis of initial physical properties, it may be because of its high initial porosity, which promotes the improvement effect of oxidative dissolution, and then leads to more obvious changes in wettability. According to the research results of Borysenko A et al. [31], it is analyzed that the main reason for the change in wettability with the action amount of oxidizing solution is that substantial oil-wet minerals such as organic matter are consumed during the oxidation process. Consequently, with the increase in the action amount of oxidizing solution, the decrease in organic matter makes the shale oil-wet minerals weaken and exhibit enhanced hydrophilicity.

**Figure 5:**
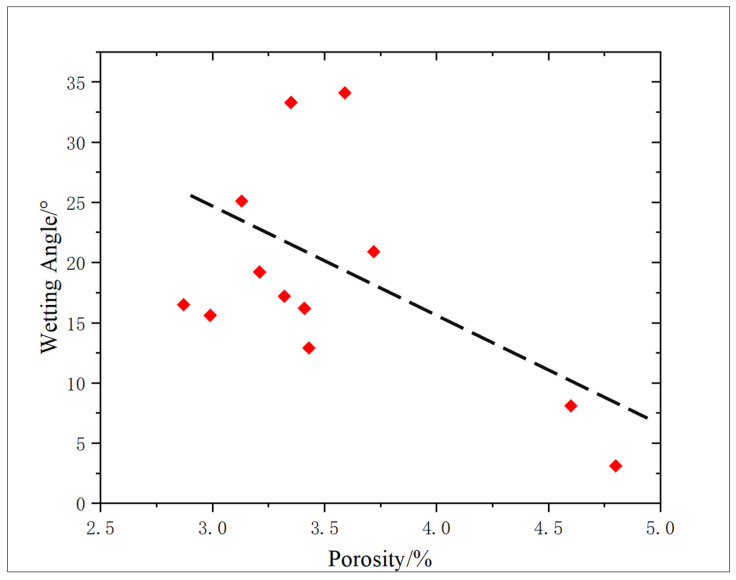
Correlation between porosity and wettability.

From the analysis in Figure 5, it indicates a discernible negative correlation between porosity and wetting angle. The reason for this phenomenon is that oxidative dissolution consumes a lot of minerals such as organic matter in the reservoir, which leads to the formation of dissolution pores, and concurrently, the decrease in oil-wet minerals increases the hydrophilicity of the core.

According to the experimental results of wettability, it can be concluded that the hydrophilicity of shale reservoirs is obviously enhanced after oxidation dissolution. With the improvement in the degree of porosity of shale reservoirs after oxidation dissolution, the spontaneous imbibition effect of shale reservoirs can be improved synergistically, and the application range of oxidation solution can be greatly increased, making it deep inside the core and not confined to the surface, and the development effect of shale oil and gas resources can be effectively improved.

### 2.3. Variation Characteristics of Shale Micropore Structure

#### 2.3.1. Evaluation of Micropore Structure of Shale Before Oxidation Dissolution

**Figure 6:**
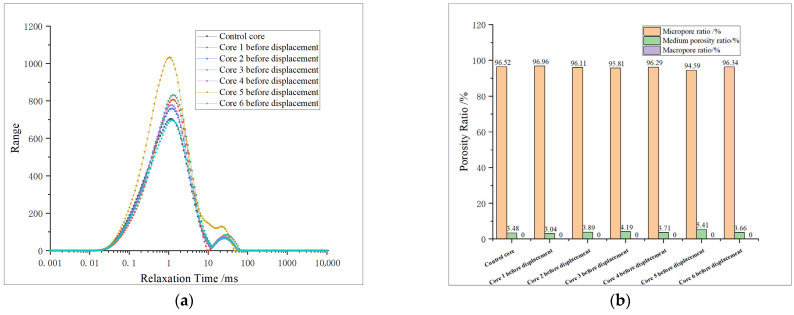
Core NMR test results before displacement: (**a**) T_2_ spectrum of core before displacement; (**b**) proportion of pores in core before displacement. Note: Macropore proportion is 0% for all samples, hence no visible purple bars.

Based on the nuclear magnetic resonance instrument, the fluid hydrogen signal in the shale core is tested, and then the distribution of nuclear magnetic resonance T_2_ spectrum of the core is obtained. According to fluid characteristics and previous test results, pores with a relaxation time of less than 10 ms are defined as micropores, those with a relaxation time of more than 10 ms and less than 100 ms as medium pores, and those with a relaxation time of more than 100 ms as macropores. The distribution of pores in different cores before oxidation dissolution is studied and analyzed, and the test results are shown in Figure 6.

In Figure 6a, the area of T_2_ spectrum curve indicates the pore volume, and the pores of different sizes can be characterized according to different relaxation times. See Figure 6b for the proportion of different pores.

According to the results of the nuclear magnetic resonance test, it can be seen that the T_2_ spectrum curves of seven cores in the study area all show a bimodal state, and micropores are mainly developed in the cores, while medium pores are less developed and macropores almost do not exist. Among them, the development degree of micropores in core No. 5 is obviously higher than in other cores, and the medium pores are obviously developed. According to the physical property test results, the porosity of this core is obviously higher than other cores, which corresponds to the nuclear magnetic resonance result curve, which proves the accuracy of the test. See Table 3 for specific experimental data.

The proportion of micropores is 94.57–96.92%, the proportion of medium pores is 3.04–5.40%, and there are almost no macropores. The distribution of reservoir pores is uniform, but almost only micropores are developed in the reservoir, resulting in extremely low porosity and permeability.

#### 2.3.2. Evaluation of Micropore Structure of Shale After Oxidative Dissolution

(1) Transverse comparison of pore change characteristics of different cores

**Figure 7:**
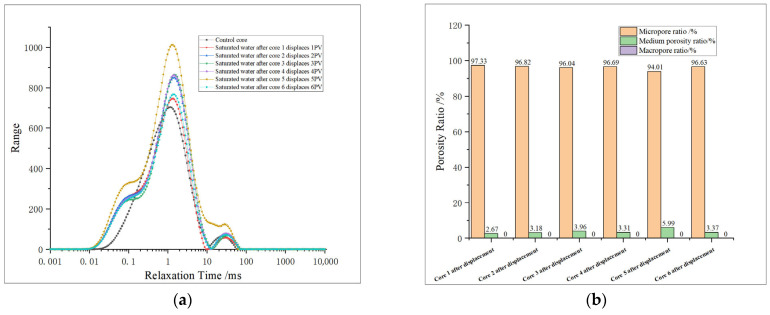
Nuclear magnetic test results of re-saturated formation water after core displacement: (**a**) T_2_ spectrum of saturated water in core after displacement; (**b**) proportion of pores in core after displacement. Note: Macropore proportion is 0% for all samples, hence no visible purple bars.

The nuclear magnetic resonance (NMR) results of re-saturated formation water after core oxidation dissolution were analyzed, and the dissolution effects of oxidizing agents under different conditions were evaluated. The pore distribution characteristics of the core after oxidation dissolution are studied in Figure 7.

From Table 4, it can be seen that the maximum pore diameters of both micropores and medium pores are increased after oxidation dissolution, but the total volume change in medium pores is relatively small, while the micropore volume is obviously increased, especially when the relaxation time is 0.01–0.1 ms, which shows that the increase in micropore volume and pore diameter is more obvious after oxidation dissolution, while the medium pore is easily blocked by sediment, resulting in a relatively small change in the total volume of medium pores.

From a pore–fracture connectivity perspective, these NMR-derived changes in pore-size distribution indicate that oxidative dissolution predominantly increases micropore volume and, at higher PV values, also partly reopens the medium-pore system, which together enhance the overall continuity of the pore network and facilitate fluid migration. Although our experiments do not resolve permeability anisotropy, the observed improvement in pore connectivity is conceptually consistent with the lamina-scale pore–fracture connectivity patterns reported by Xie et al. (2025) [6] for untreated Wufeng–Longmaxi shale, where silty laminae and their assemblages provide efficient flow pathways due to better pore–fracture linkages. In contrast to their focus on intrinsic lamina-controlled connectivity in the pristine reservoir, our results suggest that oxidative dissolution can selectively dissolve reactive components, partially remove sealing precipitates, and thereby “reactivating” or improving lamina-related pore–fracture networks. The temporary permeability and porosity minima observed around 4 PV in this study can be interpreted as an intermediate stage where oxidation-induced precipitation locally blocks pores and micro-fractures, whereas continued injection and flushing of oxidizing solution at higher PV (≥5 PV) remove sediments and lead to a net improvement in lamina-parallel pore connectivity [6].

**Figure 8:**
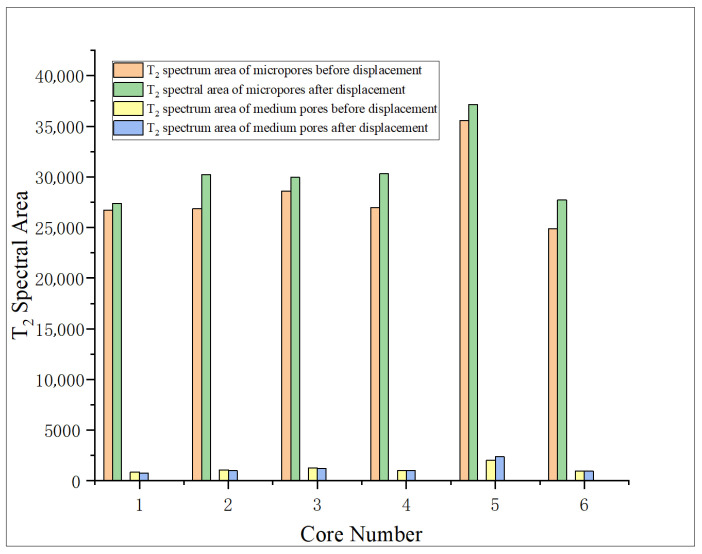
Variation characteristics of T_2_ spectrum area of shale pore before and after displacement.

From Table 5 and Figure 8, it is found that the proportion of micropores obviously increases after oxidation dissolution, with a range of about 0.22–0.71%, while the proportion of medium pores decreases in most samples, and the proportion of medium pores increases by 0.58% only at 5 PV, which proves that oxidation dissolution mainly produces micropores, but the change range of medium pores is not obvious.

The T_2_ spectrum area of the medium pore tends to decrease when displacing 1 PV. In association with the analysis of physical property test results, it is considered that the initial volume of the medium pore in the core is small, and the initial stage of oxidation dissolution is affected by dissolution and acidification, which enhances the pore connectivity of the core, but due to the short action time and the closure of some micro-cracks after displacement, the volume of medium pores is reduced. When displacing 2–3 PV, the volume of medium pores obviously decreased, indicating that plugging occurred. However, at 5 PV, the volume of medium pores began to increase, indicating that the sediment was discharged from the core, the plugging situation was relieved, and the porosity and permeability were obviously improved.

(2) Longitudinal comparison of pore variation characteristics of different cores

**Figure 9:**
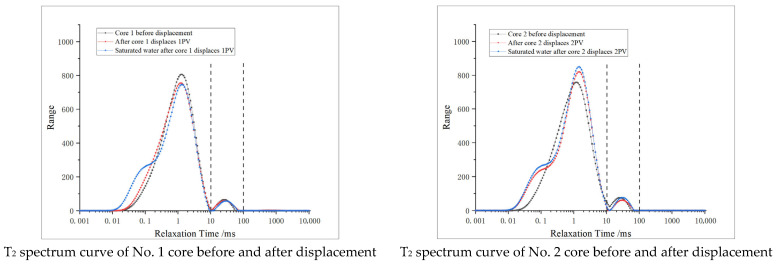

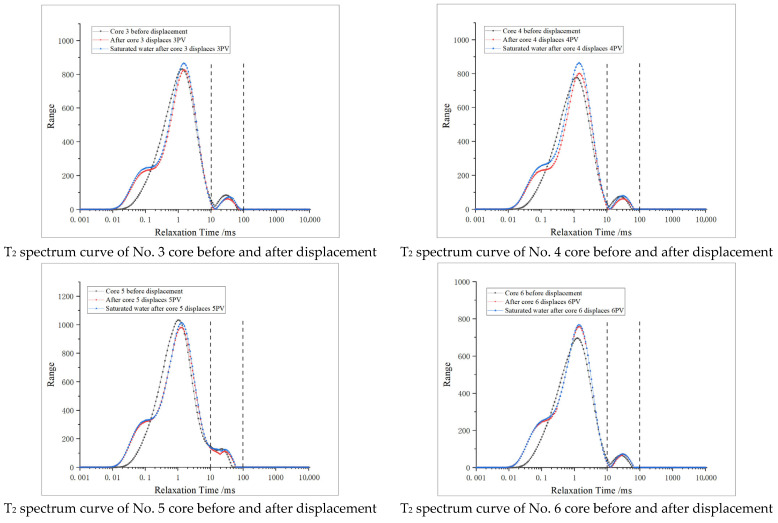
Changes in T_2_ spectrum curves of different cores before and after displacement.

The six cores are bimodal before and after displacement (Figure 9), and after displacement, micropores are generated at 0.01–0.1 ms, indicating that oxidative dissolution can generate more micropores. The T_2_ spectral curves of core 1 after displacement and core 1 after re-saturation with formation water are obviously different at 0.01–0.1 ms, and the difference in total area is small, indicating that the pore space stability is poor when the amount of oxidation solution is low. The peaks of the T_2_ spectrum for medium pores tend to shift to the right, with little change in area. The area of the T_2_ spectrum for the re-saturated water increased obviously after the core displacement of No. 2 and No. 3, while the medium pores all decreased, indicating that the medium pore is affected by sedimentation plugging. The blockage of medium pores in core No. 4 has been alleviated. The medium pores of cores No. 5 and No. 6 showed an increasing trend after the action of oxidizing solution, indicating that the medium pore volume increased and the pore size became larger. The analysis and comparison of the results after the oxidation dissolution of different cores concluded that the pore space stability of the core is poor, and the medium pore is easy to be blocked when the amount of oxidation solution is low, and the blockage is relieved when the displacement is 4 PV. With the further increase in the amount of oxidation solution (5–6 PV), a large amount of oxidation solution carries the sediment out of the core, resulting in a significant increase in the medium pore.

By comparing the effect of dissolution improvement under different amounts of oxidation solution, it is concluded that the effect of dissolution improvement is unstable when the amount of oxidation solution is low, and the pore structure still changes obviously after the oxidation dissolution, resulting in the decrease in porosity. As the amount of action increases, the effect of oxidation dissolution on the dissolution of micropores is more obvious, but it is easy to produce sediment and has a certain plugging effect on medium pores. With the continuous increase in the amount of oxidizing solution (above 5 PV), the content of easily oxidized components such as pyrite decreases, and the amount of sediment also decreases. Concurrently, a large amount of oxidizing solution can dissolve the sediment or take it out of the core, which makes the effect of medium pore plugging smaller, increases the medium pore, and improves the physical property of the reservoir.

### 2.4. Effect of Oxidative Dissolution on Mechanical Properties of Shale

**Figure 10:**
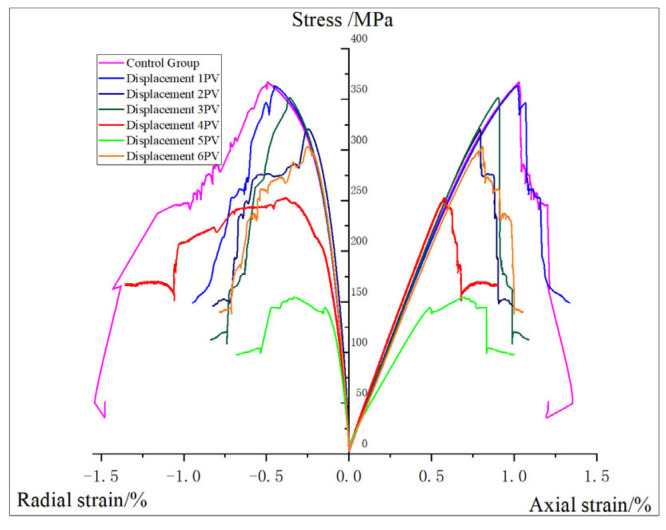
Core triaxial compression result curve.

Based on the GCTS triaxial rock testing system, the elastic mechanical parameters of the core are tested, and the experimental result curve is shown in Figure 10.

The specific experimental results are shown in Table 6. According to the selected formation depth and reservoir characteristics, the confining pressure is finally set at 20 MPa. The compressive strength of the core is between 154.85 MPa and 366.94 MPa, with an average of 301.97 MPa. The elastic modulus is between 28.33 GPa and 44.19 GPa, with an average of 39.87 GPa. Poisson’s ratio is between 0.215 and 0.343, with an average value of 0.267.

**Figure 11:**
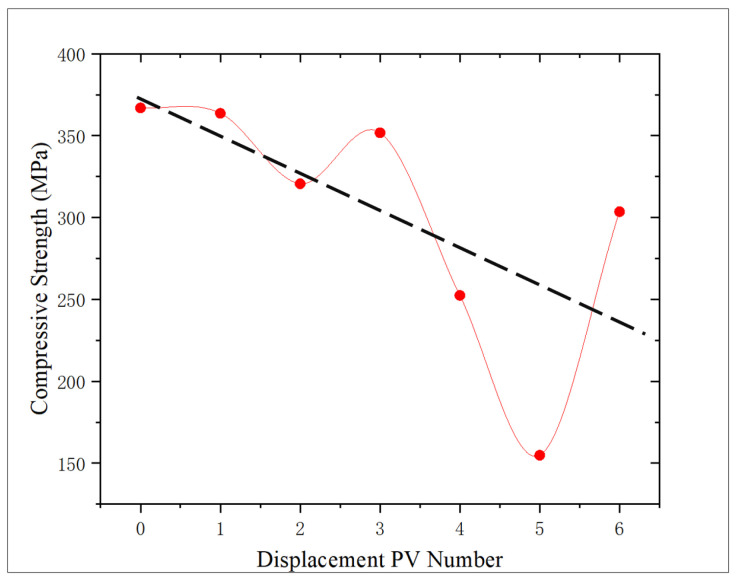
Relationship between displacement PV number and compressive strength.

According to Figure 11, the change trend of the compressive strength of the core after oxidation dissolution with the amount of oxidation solution is analyzed. Compared with the core without displacement, the compressive strength of the core shows a downward trend, decreasing by 0.89%, 12.63%, 4.15%, 31.21%, 57.80%, and 17.26%, respectively, with an average decrease of 20.66%. The sediment produced during the displacement of 3 PV may lead to shale pores being blocked, which will reduce the dissolution effect of oxidation solution and thus slightly increase the compressive strength. However, with the further increase in the amount of oxidation solution, the sediment will be discharged from the core, the dissolution effect will be further strengthened, and the compressive strength of the rock will decrease. At 5 PV, an abnormally low value is observed, and the compressive strength is reduced by 57.80% compared with the non-displaced core. The analysis is that, due to the high initial porosity and permeability of the core, the improvement effect of oxidative dissolution is significantly higher than that of other cores, and the compressive strength is significantly reduced, which proves that the initial physical properties have a significant influence on the mechanical properties after oxidative dissolution.

**Figure 12:**
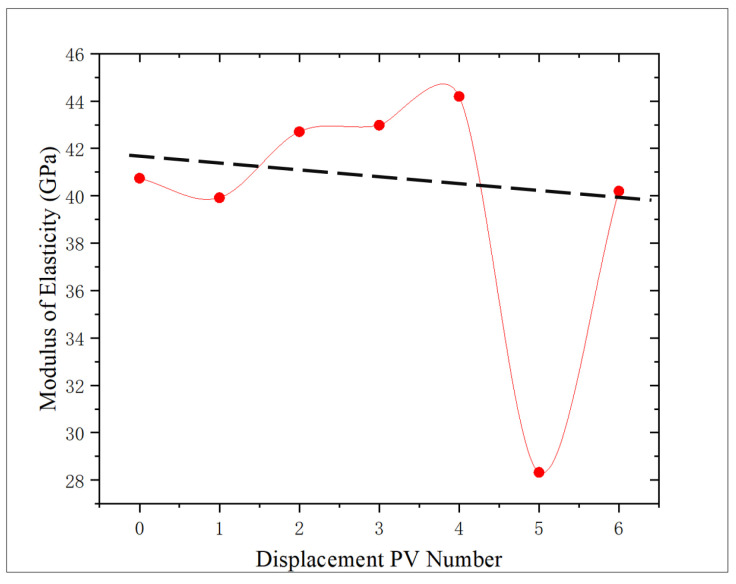
Relationship between displacement PV number and elastic modulus.

According to Figure 12, the change trend of elastic modulus of the core after oxidation dissolution with the action amount of oxidation solution is analyzed, and the change ranges are −2.02%, 4.83%, 5.49%, 8.48%, −30.46%, and −1.33%, respectively, compared with the non-displaced core. The elastic modulus of shale decreases by 2.02% when displacing 1 PV, and increases with the increase in the amount of oxidizing solution, reaching the maximum at 4 PV, which is 8.48% higher than that of the control core, which proves that the elastic modulus of the core increases with the increase in the amount of oxidizing solution. However, when the displacement multiple reaches 5–6 PV, the elastic modulus of the core decreases, indicating that the sediment is discharged from the core and the elastic modulus of the core decreases. Consistent with the test results of compressive strength, an abnormally low value at 5 PV is 30.46% lower than that of the non-displaced core, which proves that the initial physical properties have obvious influence on the oxidation dissolution effect.

**Figure 13:**
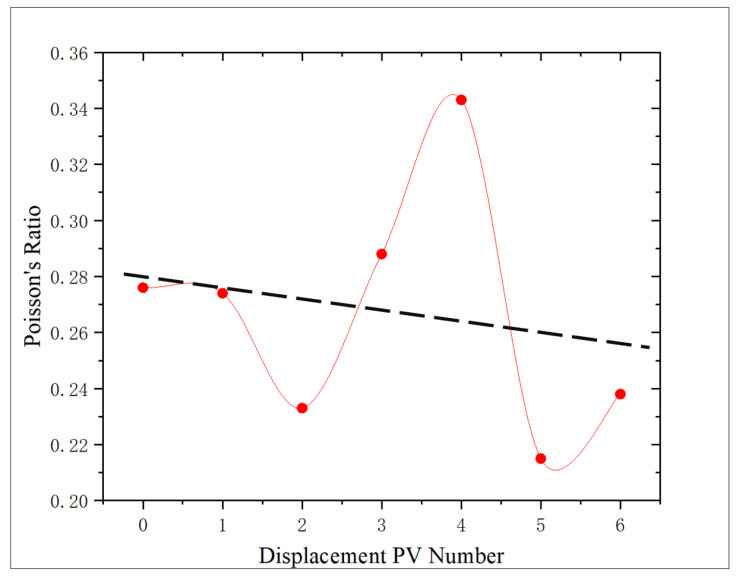
Relationship between displacement PV number and Poisson’s ratio.

According to Figure 13, the change trend of Poisson’s ratio of the core after oxidation dissolution with the amount of oxidation solution is analyzed. Compared with the control core, the change ranges are −0.72%, −15.58%, 4.35%, 24.28%, −22.10%, and −13.77%, respectively, showing the trend of first decreasing and then increasing, reaching the maximum at 4 PV and then decreasing. Poisson is larger at 4 PV, which is 24.28% higher than that of the non-displaced core, which proves that the components deposited in the core will increase the fracturing difficulty. However, with the further increase in the amount of oxidation solution, Poisson’s ratio obviously decreased, reaching the lowest value at 5 PV, which was 22.1% lower than that of the non-displaced core. It shows that a lower Poisson’s ratio can improve the fracturing effect of shale reservoir to some extent when the amount of oxidation solution is high.

The mineral- and lamina-controlled nature of oxidative dissolution also explains the observed trends in mechanical degradation and gel-breaking performance. Specimens subjected to higher degrees of carbonate and pyrite removal, as indicated by the ion-leaching tests, exhibit larger reductions in compressive strength and elastic modulus, because the preferential dissolution of load-bearing carbonate cements and pyrite-supported grain contacts reduces the effective stiffness of the lamina-bounded framework and facilitates micro-crack initiation and coalescence. At the same time, the creation of additional pore space and micro-scale connectivity inferred from the NMR data increases the accessible surface area for oxidant and gel contact, thereby accelerating oxidant-based gel-breaking reactions. Consequently, oxidants such as NaClO that strongly attack pyrite-rich laminae not only open additional fluid pathways but also act as efficient gel breakers, whereas oxidants that generate more secondary precipitates (e.g., KMnO_4_) can partially offset these benefits by locally clogging pores and hindering gel transport. This coupled “mineralogy–pore structure–mechanical strength–gel-breaking” perspective provides a useful basis for designing oxidative dissolution treatments and oxidant gel breakers in laminated, mineralogically heterogeneous shale reservoirs.

### 2.5. Relationship Between Porosity and Mechanical Properties

The pore structure and connectivity mode in the core affects the mechanical properties of the reservoir. Generally, the greater the porosity of the core, the smaller its compressive strength and elastic modulus [32]. The elastic mechanical parameters of the core are obtained by experiments, and then the relationship model between the pore and mechanics is established based on the experimental results of core physical properties, and the interaction mechanism between pore and mechanics is analyzed, to achieve a more accurate evaluation of reservoir properties.

**Figure 14:**
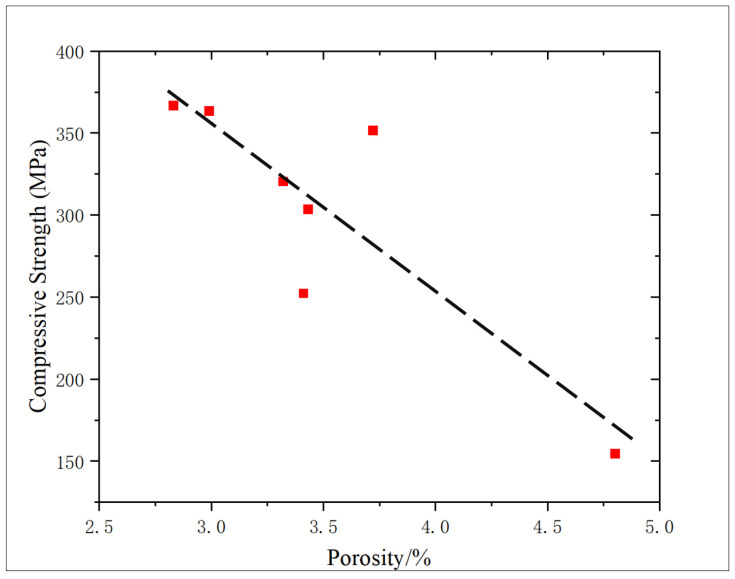
Correlation analysis of porosity and compressive strength.

From Figure 14, the correlation between porosity and compressive strength is analyzed. With the increase in porosity, the compressive strength of the core shows a downward trend, and the correlation is 0.7424, indicating a good correlation. It is proved that the increase in porosity in the process of oxidation dissolution reduces the degree of cementation of the core, and the dissolution of the filling material in the core leads to the decrease in compressive strength, and it is proved that the porosity increase in the later period of oxidation solution decreases the core strength.

**Figure 15:**
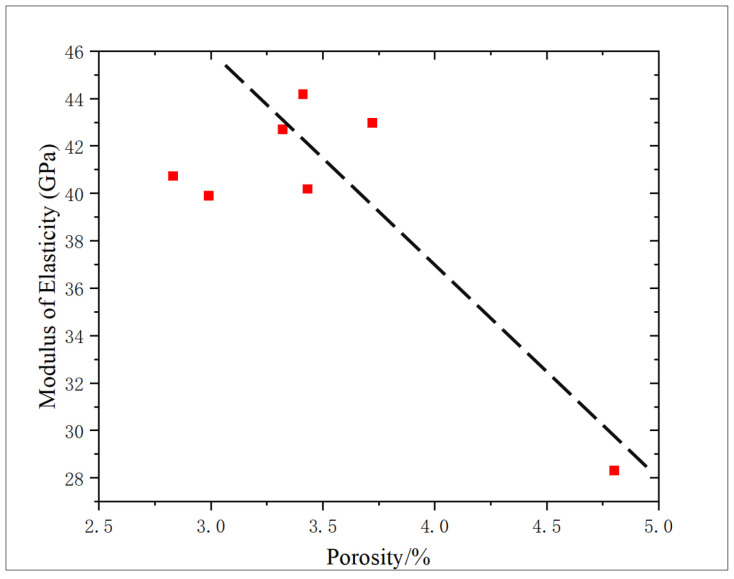
Correlation analysis of porosity and elastic modulus.

The correlation between porosity and elastic modulus is 0.5912, showing a negative correlation (Figure 15), which is consistent with the change trend of compressive strength, showing that with the increase in porosity, the elastic modulus of core decreases, which proves that there is an interaction mechanism between porosity and core strength characteristics in shale reservoirs.

**Figure 16:**
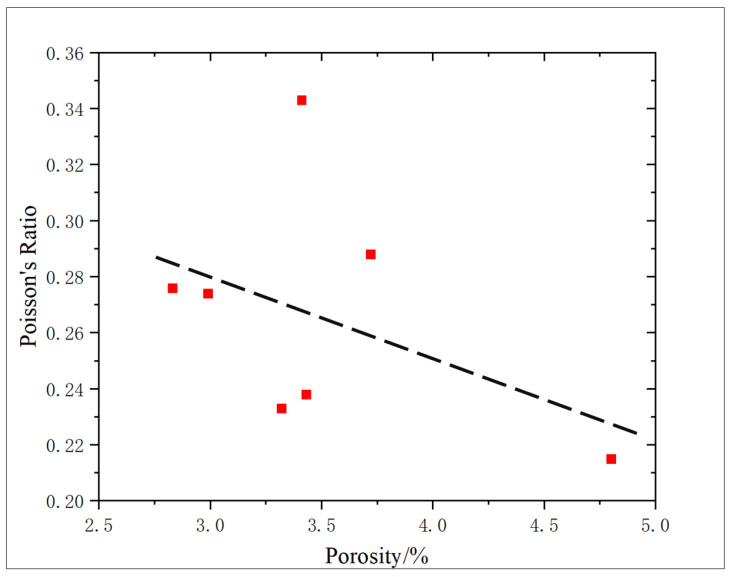
Correlation analysis of porosity and Poisson’s ratio.

The correlation between porosity and Poisson’s ratio is poor. The variation curve of Poisson’s ratio and the action amount of oxidation solution also show that the regularity of Poisson’s ratio is poor (Figure 16). Porosity changes include porosity increase caused by dissolution and porosity decrease caused by sediments, and the content of sediments affects Poisson’s ratio to some extent, so there are some differences in Poisson’s ratios of the cores with the same porosity, which lead to a poor correlation between Poisson’s ratio and porosity.

### 2.6. Discussion

Overall, the oxidative dissolution trends observed in this study are broadly consistent with recent research on shale–oxidant reaction kinetics and oxidative stimulation in heterogeneous reservoirs. Kinetic tests on Longmaxi and other organic-rich shales have shown that oxidants such as H_2_O_2_ promote the progressive removal of organic matter and pyrite, accompanied by transient pH drops and the widening of pre-existing fractures, which in turn reduces stress sensitivity and facilitates unpropped fracture conductivity [9,14]. Our triaxial results similarly indicate that increasing oxidant exposure (PV) leads to systematic reductions in compressive strength and elastic modulus, particularly once dissolution products and fines are flushed out of the pore network. Meanwhile, recent gel-fracturing studies in heterogeneous reservoirs have highlighted that gel design and gel-breaking strategies must account for spatial variations in mineralogy, permeability, and fracture connectivity, because these heterogeneities control both oxidant transport and gel degradation [33,34]. By explicitly linking oxidant-induced changes in pore structure and mechanical properties to gel-breaking performance, this study extends these concepts to a coupled “rock–fluid–gel” framework, which can be used to optimize oxidative gel-breaker dosage and contact time in laminated, mineralogically heterogeneous shale reservoirs.

From a mechanistic perspective, the main experimental observations in this study can be explained by the coupled effects of mineral-selective dissolution, secondary precipitation, and lamina-controlled heterogeneity. At low PV numbers, NaClO preferentially oxidizes organic matter and pyrite and partly dissolves carbonate cements, generating dissolution pores and gas that open micro-fractures and increase permeability and porosity. With increasing oxidant dosage, a prolonged reaction in an alkaline environment favors the formation of ferric hydroxides and other precipitates and mobilizes fines, so that these reaction products temporarily accumulate in pore throats and lamina-bounded micro-fractures, leading to the observed minimum in permeability and porosity around 4 PV. When the injected volume exceeds this threshold, continued dissolution and the flushing action of the flowing oxidant progressively remove precipitates and fines, and matrix dissolution becomes dominant again, causing permeability and porosity to resume increasing. At the same time, the consumption of oil-wet organic matter and the exposure of more quartz- and clay-dominated surfaces improve water wetness, explaining the strong negative correlation between porosity and contact angle. The dissolution of load-bearing carbonate cements and pyrite-supported grain contacts reduces compressive strength and elastic modulus, consistent with the negative porosity–mechanics correlations, while the creation of additional pore space and micro-scale connectivity enhances oxidant penetration and contact with fracturing gel residues. These coupled mechanisms clarify why NaClO-based oxidative dissolution can simultaneously enlarge flow pathways, improve hydrophilicity, and enhance gel-breaking efficiency, as well as highlighting the need to optimize oxidant dosage and contact time to avoid the excessive weakening of the shale matrix.

**Figure 17:**
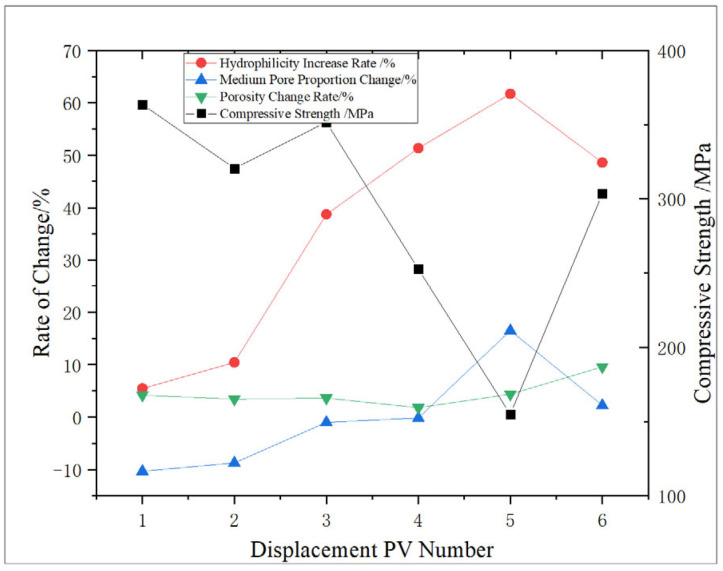
Variation trend of shale properties with the increase in oxidation solution.

Experimental results show that, with the increase in the action amount of oxidizing solution, the dissolution degree of pyrite, organic matter, and other minerals in shale increases, and the porosity and permeability of shale show an increasing trend. The reduction in oil-wet minerals such as organic matter enhances the hydrophilicity of shale. Concurrently, due to the dissolution of some minerals, the degree of shale cementation decreases, and the rock strength shows a downward trend (Figure 17). According to the research results and the influence degree of different parameters on the oxidation dissolution rate, the characterization model of oxidation dissolution reaction rate and the main influencing factors of shale reservoir are constructed.

(1) $$N=0.02w\left(-0.1t+3.86\right)(0.7032v^{2}-4.1245v+8.262)$$

where *N*—the reaction rate, %; *w*—the mass concentration of oxidation solution, %; *t*—the reaction time, h; *v*—the displacement fluid volume, PV.

With the further increase in the action amount of oxidizing solution, some iron hydroxide precipitates may be produced to block the reservoir, which reduces the efficiency of oxidation dissolution, and then lead to the decrease in the increase degree of reservoir porosity and a slight increase in strength. When the action amount reaches 5–6 PV, the oxidation solution transport sediments away from the core, significantly increasing both porosity and permeability while reducing rock strength.

Analysis of the shale micropore structure reveals that the proportion and pore diameter of micropores in shale obviously increase after oxidation dissolution, while the change range of medium pores is relatively minor due to the joint action of dissolution and sediment blockage. When the amount of oxidation solution is large (5–6 PV), the sediment is discharged from the pores, and the medium pores obviously increase. The area of the T_2_ spectrum for medium pores shows that medium pores have an obvious positive correlation with the amount of oxidation solution, and the correlation coefficient is 0.6022. Moreover, the medium porosity has a significant influence on the shale strength, showing a strong negative correlation between the two, and the correlation coefficient is 0.778, which indicates that with the increase in the action amount of oxidation solution, the medium porosity increases and the rock strength decreases, which can obviously improve the reservoir physical properties and is conducive to the expansion and extension of fractures.

Recent micro-CT and SEM studies on Wufeng–Longmaxi shales have provided direct visual evidence for how pore networks and micro-fractures evolve at the lamina and grain scale during fluid–rock interaction [35]. The NMR-derived pore-size evolution and the observed changes in permeability and mechanical properties are broadly consistent with these imaging results. Specifically, the preferential enlargement of micropores and the delayed enhancement of medium-pore connectivity inferred from our NMR data agree with previously reported observations of dissolution channels and secondary pores developing along mineral and lamina boundaries in Wufeng–Longmaxi shales. This correspondence supports the interpretation that NaClO oxidation preferentially attacks reactive minerals (such as pyrite and certain carbonates) and organic matter, generates additional dissolution pores, and modifies existing pore throat structures, which together contribute to the observed permeability increase and strength reduction.

The oxidation solution demonstrates robust dissolving capabilities towards organic matter and pyrite present within shale matrices. When the action amount of oxidation solution increases, the hydrophilicity of shale increases and the porosity generally increases, and there is a certain negative correlation between porosity and wetting angle. The increase in porosity leads to the decrease in shale cementation degree, which leads to a general downward trend of shale strength. Porosity has a strong negative correlation with rock strength, in which the correlation coefficient of porosity–compressive strength is 0.7424 and porosity–elastic modulus is 0.5912. The synergistic effect of various factors can effectively improve the physical properties of shale reservoirs, reduce the difficulty of oil and gas mobility, and improve the development effect of shale oil and gas resources.

**Figure 18:**
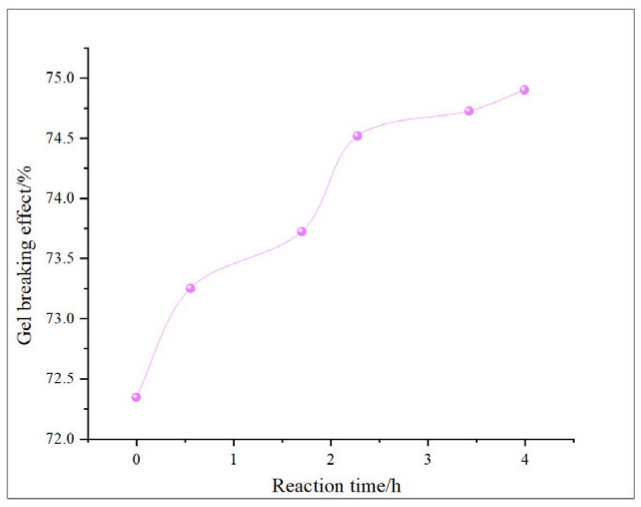
Relationship between NaClO reaction time and gel-breaking effect.

**Figure 19:**
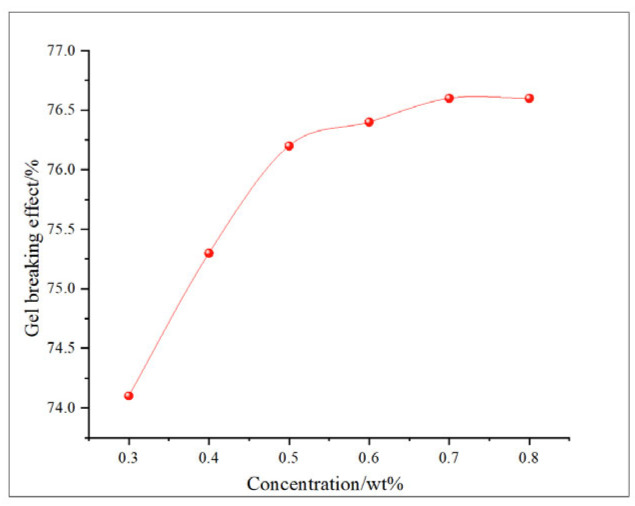
Relationship between NaClO concentration and gel-breaking effect.

**Figure 20:**
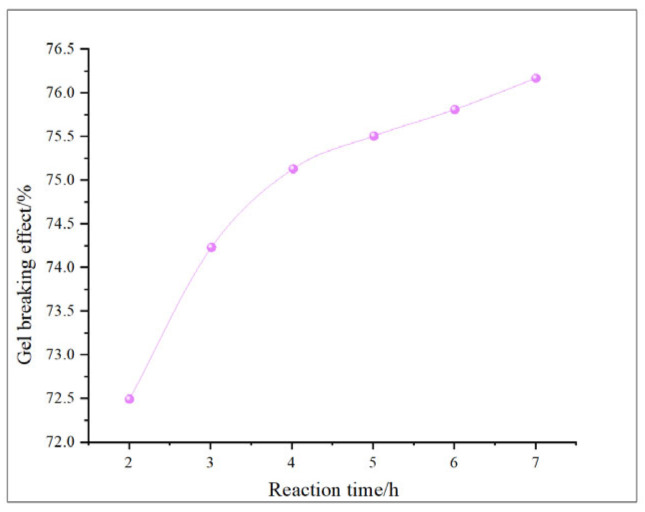
Relationship between H_2_O_2_ reaction time and gel-breaking effect.

**Figure 21:**
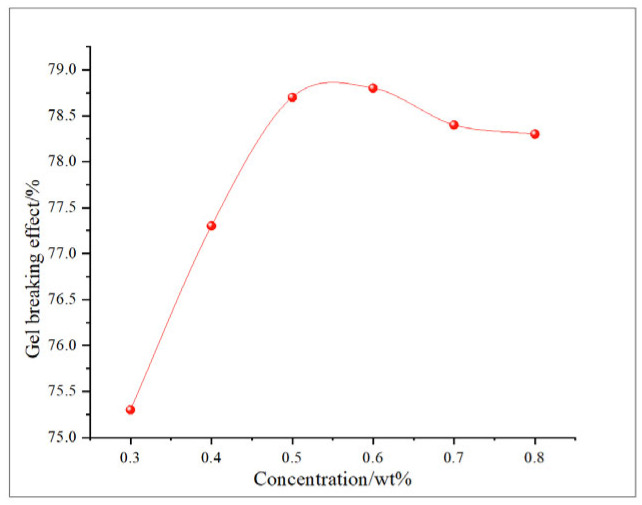
Relationship between H_2_O_2_ concentration and gel-breaking effect.

**Figure 22:**
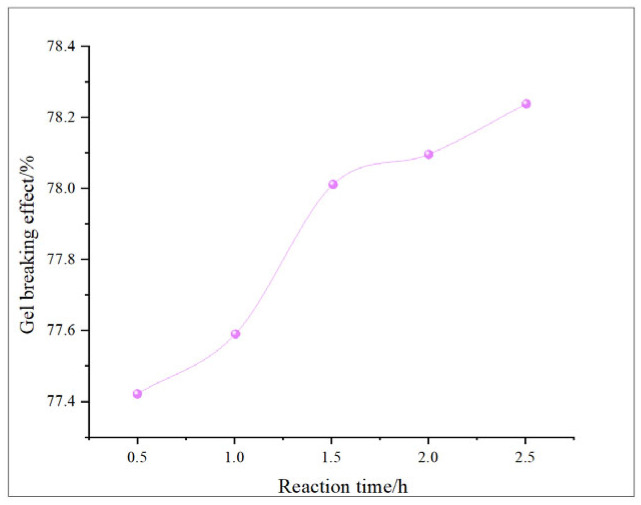
Relationship between KMnO_4_; reaction time and gel-breaking effect.

**Figure 23:**
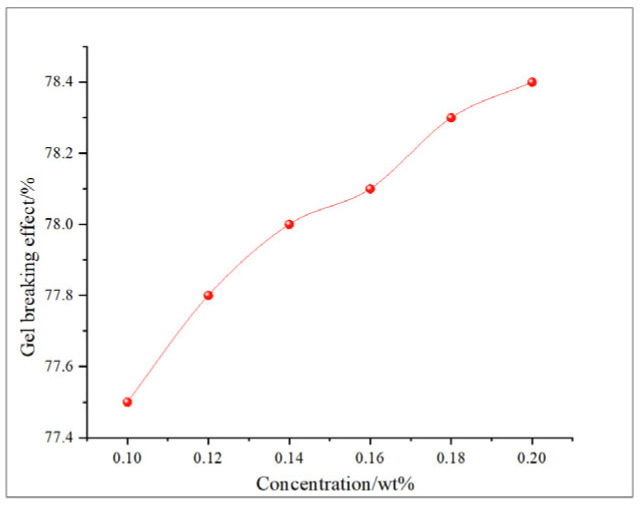
Relationship between KMnO_4_ concentration and gel-breaking effect.

This study comprehensively discusses the effects of different oxidant concentrations and reaction times on the gel-breaking efficiency of fracturing fluids through the comparative analysis of existing research. The gel-breaking performance of NaClO is positively correlated with its concentration; when the concentration reaches 0.6 wt%, the improvement in gel-breaking efficiency tends to plateau at 76.4%. The gel-breaking effect increases with the extension of reaction time and enters a plateau phase after 2.5 h, with the gel-breaking efficiency reaching 74.5%, so the optimal reaction time for NaClO is determined as 2.5 h (Figure 18 and Figure 19). As the concentration of H_2_O_2_ increases, the gel-breaking efficiency improves correspondingly, but when the concentration exceeds 0.5 wt%, the growth rate slows down significantly, with the gel-breaking efficiency reaching 78.7%. This phenomenon may be related to the side reaction between ·OH free radicals and H_2_O_2_. Under high-H_2_O_2_ concentration conditions, this reaction competitively consumes active free radicals, leading to reduced oxidation efficiency and the ineffective decomposition of H_2_O_2_. The reaction kinetics presents a “delay–acceleration–moderation” characteristic: the change is not significant in the first 0–2 h, it rises rapidly between 3 and 4 h, and it slows down when the time exceeds 5 h. Therefore, the suitable reaction time is set at 5 h, with the gel-breaking efficiency reaching 75.5%. It should be noted that the reaction rate is relatively slow and oxidation efficiency is limited in a catalyst-free system, so H_2_O_2_ should be used with catalyst activation in practical applications (Figure 20 and Figure 21). As a strong oxidant, the gel-breaking efficiency of KMnO_4_ increases with concentration; when the concentration reaches 0.1 wt%, the gel-breaking efficiency reaches 77.5%, and further increasing the concentration yields limited improvement in gel-breaking effect. The time effect is relatively moderate, as a good treatment level (77.42%) can be achieved within 0.5 h (Figure 22 and Figure 23) [36,37].

A comprehensive comparison of the gel-breaking efficiency of the three oxidants over time shows that NaClO achieves the best gel-breaking effect (74.52%) at 2.5 h, making it an efficient acidic gel breaker. H_2_O_2_ requires a longer reaction time, reaching 75.5% at 5 h, and usually needs to be combined with a catalyst to activate its oxidation capacity, resulting in relatively higher operational costs and complexity. The greatest advantage of KMnO_4_ lies in its extremely rapid reaction rate, as it can reach 77.42% gel-breaking efficiency within 0.5 h. Therefore, based on the research results, NaClO can be adopted when only oxidative dissolution stimulation is required for shale reservoirs without subsequent fracturing operations, as it can achieve good oxidation effects with little impact on other working fluids. When fracturing processes are needed after oxidation, KMnO_4_, H_2_O_2_, and other oxidants can be considered according to the actual reservoir characteristics. Under the condition of ensuring oxidative dissolution efficiency, these oxidants can also achieve good gel-breaking effects, thereby improving the oxidative dissolution stimulation effect of the reservoir [38].

After oxidation dissolution, the physical properties of shale are obviously improved, and the increase in porosity and permeability greatly increases the application range of oxidation solution. Moreover, the oxidation dissolution of organic matter is conducive to enhancing the hydrophilicity of shale, and the improvement in porosity and permeability can jointly improve the development effect of imbibition. Additionally, oxidative dissolution can not only enhance gel-breaking efficiency to a certain extent but also change the mechanical properties of shale, broaden the extension range of fractures, and form self-support to improve the conductivity. It should be noted that the batch oxidative dissolution experiments were conducted in unbuffered solutions and the instantaneous pH evolution was not monitored; therefore, the detailed coupling between pH transients and reaction rates could not be quantified in this study. Future research will combine in situ pH monitoring and kinetic modeling to further constrain shale–oxidant reaction mechanisms. Based on the experimental results, a dynamic model of enhanced imbibition through oxidation dissolution has been developed for evaluating the multi-field effects of oxidation–imbibition and mechanical damage, providing a feasible tool for expanding the range, mode, and control method of oxidation solution during hydraulic fracturing. A new method and technology for shale gas exploitation under the coupling effect of multi-field mutual feedback is developed.

## 3. Conclusions

Although the experiments in this study are conducted on organic-rich marine shales from the Wufeng Formation in the Sichuan Basin, the controlling processes we identify—the oxidation of organic matter and pyrite, the dissolution and rearrangement of brittle minerals, and the associated evolution of pore structure, permeability, wettability, and mechanical properties—are common to many shale reservoirs with comparable mineralogy and maturity. Therefore, the qualitative trends between oxidant dosage, porosity/permeability enhancement, and mechanical weakening are expected to be applicable to other quartz- and carbonate-rich, high-TOC shales. Nevertheless, the quantitative parameters (e.g., critical PV corresponding to permeability minima and the extent of strength reduction) must be recalibrated for specific formations, especially for clay-rich or structurally deformed shales, and this represents a key direction for future research. The major conclusions drawn from this study are as follows:

1. The dissolution effect of three commonly utilized oxidants, KMnO_4_, NaClO, and H_2_O_2_, on shale was evaluated. The oxidant 10 wt% NaClO had the best dissolution effect on pyrite. After oxidation dissolution, the permeability of shale increased by 66.67–266.67%, and the porosity increased by 1.79–9.58%. Micropores predominated within shale samples (94.57–96.92%), whereas medium pores constituted only 3.04–5.40%.

2. Shale samples from this study exhibit hydrophilic properties that improve significantly—from 5.45% to 61.73%—with increasing amounts of oxidation solution applied. It is analyzed that the hydrophilicity of shale is enhanced due to the large amount of oil-wet minerals such as organic matter being consumed. Concurrently, due to the increase in solution pores, the core porosity and wetting angle show a negative correlation. The improvement in hydrophilicity and porosity can cooperatively improve the spontaneous imbibition effect of shale reservoirs, so that the oxidation solution goes deep into the reservoir, and the application range of the oxidation solution is greatly increased.

3. With that increase in the action amount of oxidizing solution, the dissolution of organic matter and other minerals and cements increases the porosity of the core, and the compressive strength shows a downward trend, and the decrease rate is the largest at 5 PV, reaching 57.8%. The elastic modulus first increased and then decreased, with a range of −30.46–8.48%. Shale porosity is negatively correlated with compressive strength and elastic modulus.

4. Shale porosity and strength, porosity, and wetting angle show a strong negative correlation after oxidative dissolution. Based on the experimental results, a dynamic model of enhanced imbibition through oxidation dissolution has been developed for evaluating the multi-field effects of oxidation–imbibition and mechanical damage, providing a feasible tool for expanding the range, mode, and control method of oxidation solution during hydraulic fracturing, and then a new method and technology for shale gas exploitation under the coupling effect of multi-field mutual feedback is developed.

Scalability: From a field-scale perspective, the proposed oxidation-assisted stimulation method offers both opportunities and challenges. On the benefit side, NaClO-induced oxidative dissolution can increase shale porosity and permeability, enhance hydrophilicity, and improve gel-breaking performance, which together have the potential to extend the effective action range of fracturing fluids, promote gas desorption, and facilitate post-fracturing clean-up in shale gas reservoirs. However, the translation of these core-scale results to heterogeneous field conditions is not straightforward. In practice, the uniform placement of oxidizing solutions, the control of reaction rate and contact time, and the management of by-products (e.g., dissolved ions and fines) remain key challenges. In addition, excessive oxidation may over-weaken the rock matrix and adversely affect wellbore and fracture stability, while the use of strong oxidants introduces extra requirements for chemical handling, corrosion control, and environmental management. Therefore, oxidation-assisted stimulation should be carefully optimized and supported by reservoir-scale simulation and staged field pilots to balance the benefits of improved reservoir properties against the operational risks and costs in full-scale applications.

## 4. Materials and Methods

### 4.1. Core Samples and Experimental Fluids

**Figure 24:**
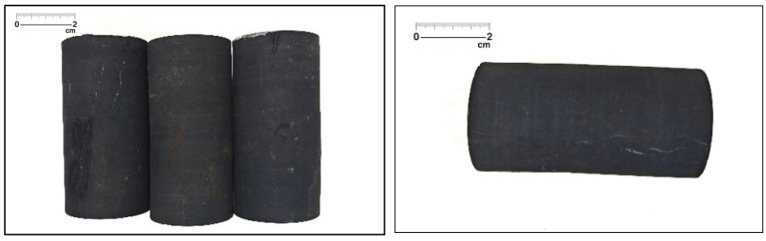
Shale core from Well ZX-5 (3013.8 m, Wufeng–Longmaxi Formation).

Shale core samples were taken from the Wufeng Formation in the Sichuan Basin, and the prepared cores were 25 mm in diameter and 50 mm in length (Figure 24). A 3% KCl solution with a salinity of 30,000 mg/L was selected to simulate formation water, and the porosity experiment was carried out after the formation water was purified. According to previously published whole-rock XRD analyses of Wufeng–Longmaxi shales in the southern Sichuan Basin, quartz accounts for ca. 33–68 wt% (average ≈ 49 wt%), clay minerals for 17–44 wt% (average ≈ 29 wt%), feldspars for 2–12 wt%, carbonate minerals for 4–36 wt% (average ≈ 12 wt%), and disseminated pyrite for ca. 2–6 wt% [39].

The simulated formation water was used to prepare oxidation solutions with different concentrations, and the properties of the three oxidants were compared. The concentration of H_2_O_2_ was 10 wt%, 15 wt%, and 20 wt%, respectively, and the selection of this concentration was mainly based on the research results of scholars such as Cheng et al. [9] and Yang et al. [21]. The NaClO concentrations were 6 wt%, 8 wt%, and 10 wt%, respectively. Because the oxidizability of KMnO_4_ is lower than NaClO—and considering experimental feasibility—its concentrations were established at 1 wt%, 2 wt%, and 4 wt%.

The physical property test results show that the permeability of shale cores is between 0.0003 and 0.0397 mD, with an average value of 0.0061 mD. The permeability is mostly less than 0.001 mD and more than 0.0001 mD, and only the permeability of No. 5 core is more than 0.01 mD, which is inferred to be caused by the development of micro-fractures in the core. The reservoir permeability in the study area is extremely low, which belongs to a ultra-low permeability reservoir. However, the porosity ranges from 2.83% to 4.60%, with an average value of 3.37%. The porosity distribution is relatively uniform compared with the permeability, and the pore throat connectivity of the reservoir is poor (Table 7).

### 4.2. Experimental Procedure

1. Permeability Testing: The permeability of the core is measured by nitrogen, and the inlet pressure is estimated to be 4 MPa according to the physical properties of the core, with confining pressure consistently maintained at 1.5 MPa to 2 MPa above inlet pressure.

2. Core Porosity Testing: The pressure in the instrument is pumped to a vacuum state of −0.1 MPa for 4 h through a core saturation device, formation water is added to completely submerge the core, and it is pressurized to 25 MPa and saturated for 24 h. The porosity of the core can be obtained according to the dry weight of the core and the wet weight after completely saturating the formation water.

3. Optimization of oxidant: For each oxidant, batch oxidative dissolution experiments were conducted on crushed shale. Specifically, 2.0 g of dry shale powder was placed in a 50 mL glass container, to which 25 mL of the oxidizing solution at the target concentration was added. This corresponds to a fixed fluid/rock ratio of 12.5 mL/g, which is within the range commonly used in oxidative dissolution studies on organic-rich shales. Based on the concentration ranges defined in Section 4.1, three concentrations were tested for each oxidant: H_2_O_2_ at 10, 15, and 20 wt%, NaClO at 6, 8, and 10 wt%, and KMnO_4_ at 1, 2, and 4 wt%. No external buffer was added, so the solution pH was allowed to evolve freely as oxidation and dissolution proceeded. To minimize evaporative loss and gas exchange with air, the container mouths were tightly covered with plastic wrap, and all containers were placed in a thermostatic water bath maintained at 80 °C. The system was kept under static conditions (no mechanical stirring), and reaction products were sampled at 12 h, 24 h, and 48 h to evaluate the time-dependent oxidative dissolution behavior.

4. Evaluation methodology: The solution after the reaction was filtered through filter paper, and the shale sample after the reaction was obtained. The ionic concentrations of Ca^2+^, Mg^2+^, Al^3+^, and SO_4_^2−^ in the filtrate were analyzed and determined by plasma emission spectroscopy and ion chromatography. By analyzing the changes in shale quality before and after the reaction, the oxidation dissolution effect was evaluated, and then the types and concentrations of oxidants were optimized.

5. Preparation of oxidizing solutions: According to the oxidant and oxidant concentration selected in the experiment, it is added into the simulated formation water and extracted and purified to prepare the oxidation solution for the experiment.

6. Displacement of oxidizing solutions: The initial flow rate is predicted according to the results of gas permeability measurement in the early stage, and the dissolution effect of oxidation solution under different action amounts is evaluated by a high-temperature and high-pressure displacement device.

7. Based on nuclear magnetic resonance (NMR) technology [40], contact-angle measurements [41] and triaxial compression tests [42] were carried out to characterize the evolution of pore structure, wettability, and mechanical properties before and after oxidative dissolution. The contact angles were measured using a standard optical contact-angle meter equipped with a built-in fitting algorithm. During each test, deionized water droplets of constant volume were gently placed on freshly cut shale surfaces, and at least five images were recorded and analyzed for each surface; the reported contact angle corresponds to the average of multiple measurements in order to reduce random errors. The imaging conditions (camera position and illumination) were kept as consistent as possible throughout the tests.

**Figure 25:**
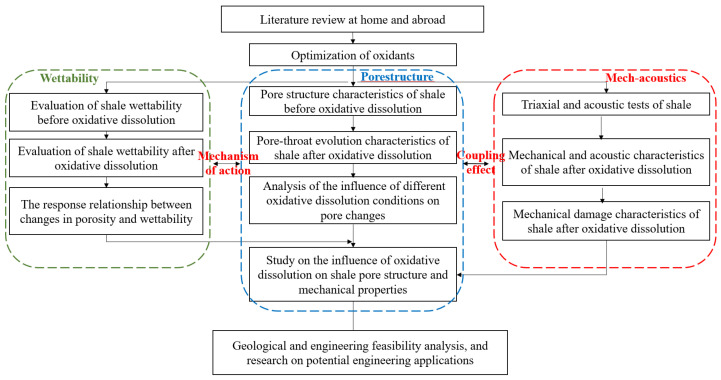
Flowchart summarizing the experimental and analytical procedure adopted in this study.

To provide a concise overview of the methodology, the main steps of this work—including core selection and basic characterization, oxidant screening, and pre- and post-treatment pore/wettability/mechanical tests—are summarized in the flowchart shown in Figure 25.

### 4.3. Oxidant Selection

The dissolution effects of three commonly used oxidants, NaClO, KMnO_4_, and H_2_O_2_, on shale reservoirs were evaluated, and the best oxidant and concentration for shale dissolve in the study area were selected.

#### 4.3.1. Changes in Core Quality After Shale Reacts with Different Oxidants

The weight of shale before and after oxidation dissolution was measured, and the change rate of shale weight before and after oxidation dissolution was calculated and analyzed using Equation (2). The results are shown in Table 8. When the weight change rate is positive, it means that the core weight decreases after the reaction, indicating that the core has been subjected to strong oxidative dissolution; however, when the weight change rate is negative, it means that the core weight increases after the reaction, indicating that after the action of oxidant, partial sediment is produced, which leads to the increase in shale weight after the reaction, and the oxidative dissolution may lead to the blockage of reservoir pores, which proves that the oxidative dissolution effect is poor.

(2) $$M\%=\frac{m_{1}-m_{2}}{m_{1}}\times 100$$

where *M* is the change rate of shale weight, %; *m*_1_ is the shale weight before oxidation dissolution, g; *m*_2_ is the shale weight of shale after oxidation dissolution, g.

After KMnO_4_ solution reacts with shale, the weight of shale increases, indicating that the reaction produces sediments. In contrast, both NaClO and H_2_O_2_ dissolve shale and reduce the weight of shale. NaClO with a weight concentration of 8 wt% and a time of 24 h has the best dissolution effect on shale, and the weight change rate of 3.92% is better than that of H_2_O_2_ with 2.13%.

#### 4.3.2. Changes in Ion Concentration in Solution After Shale Reacts with Different Oxidants

According to the reaction formula [21] obtained by consulting the literature and the research results of relevant scholars, the possible main ions (Ca^2+^, Mg^2+^, Al^3+^) are tested, and the results are shown in Table 9. Iron in shale mainly comes from pyrite, and the reaction amount of Fe^2+^ can be characterized by measuring sulfate ions.

After the reaction of H_2_O_2_ oxidation solution, the concentrations of Ca^2+^ and Mg^2+^ ions are obviously higher than those of the other two oxidant solutions, indicating that it has a better dissolution effect on carbonate minerals such as quartz. After NaClO oxidation solution reacts with shale, the concentration of SO_4_^2−^ ion is obviously higher than that of KMnO_4_ and H_2_O_2_, and it increases with the increase in solution concentration and action time, indicating that NaClO has the best dissolution effect on pyrite. In association with the results of shale weight change rate, the weight change rate at 24 h is obviously higher than that at 12 h and 48 h, while the weight change rate of NaClO with 6 wt% is the lowest at 24 h, and there is little difference between the weight change rates of 8 wt% and 10 wt%. In association with the ion concentration results of 24 h and 10 wt%, the ion concentration tested is obviously higher than that of 6 wt% and 8 wt%. In addition, the oxidant NaClO has a good effect as a plugging remover and a gel breaker in the field [38,43], so 10 wt% NaClO was selected as the oxidant for the experiment.

## Figures and Tables

**Table 1:** Comparative analysis of oxidative stimulation in Wufeng–Longmaxi shale: previous studies vs. this study.

Study	Key Findings (Very Brief)	Relation to This Work/Novelty Gap
Kuila U et al. (2014) [6,7,8]	Compared oxidant-driven removal of organic matter from shales with different mineralogy and TOC, showing that mineral composition and OM content strongly control oxidative dissolution efficiency and verifying the feasibility of oxidative stimulation.	Moves beyond feasibility tests on different blocks to a systematic, PV-dependent NaClO treatment on Wufeng shale cores under unified conditions, quantitatively tracking how oxidative dissolution modifies pore structure, permeability, and wettability.
Cheng Q et al. (2021) [9]	Investigated H_2_O_2_ oxidative dissolution of Longmaxi organic-rich shale; combined kinetics on crushed/sliced samples (2–10 wt%, 40–80 °C) with stress-sensitivity tests on fractured plugs; quantified OM removal and Arrhenius-type rates; showed oxidation widens fractures and reduces stress sensitivity, helping maintain unpropped fracture conductivity.	Extends from H_2_O_2_–Longmaxi systems to a NaClO-dominated treatment of Wufeng shale, and in addition to kinetics-type insight, integrates core-scale NMR pore structure evolution, contact-angle wettability tests, and triaxial mechanical measurements into a single evaluation framework.
Cheng Q et al. (2020) [13]	Tested shale wettability and spontaneous imbibition after oxidation; found that oxidative dissolution enlarges pore radius, greatly shortens imbibition time, and increases imbibition capacity, alleviating water lock and enhancing imbibition-driven recovery.	Goes beyond post-treatment wettability tests by quantitatively linking NMR-derived pore-size distribution, porosity, and contact angle to changes in strength and elastic parameters, and embedding these relationships into a dynamic oxidation-enhanced imbibition model.
Yang S et al. (2020) [21,22]	Evaluated H_2_O_2_, NaClO, and Na_2_S_2_O_8_ oxidants for Wufeng shale; found that acidic oxidants predominantly dissolve carbonate minerals, whereas alkaline oxidants focus more on aluminosilicate components; gas generated during oxidation can supplement formation energy and help create and extend fractures; NaClO primarily targets OM and pyrite, effectively increasing pore volume and radius, while Na_2_S_2_O_8_ mainly decomposes carbonates and also alters surface functional groups and wettability, enhancing spontaneous imbibition and hydrocarbon mobility.	Builds on their multi-oxidant comparison by (i) selecting NaClO as the primary oxidant for Wufeng shale, (ii) systematically varying PV to capture the non-monotonic evolution of porosity/permeability, (iii) coupling NMR, contact-angle, permeability/porosity, and triaxial tests, and (iv) jointly assessing oxidant gel-breaking performance, thus yielding directly applicable design guidance for oxidation-assisted fracturing.
Liang X et al. (2021) [23,24,25]	Investigated acid and oxidative reactions between Na_2_S_2_O_8_ and Marcellus shale; concluded that dissolution involves both acid dissolution and oxidation, with acid dissolution dominating; dissolution of calcite and dolomite was more pronounced than pyrite oxidation.	Shifts from Na_2_S_2_O_8_–Marcellus systems, where carbonate dissolution dominates, to NaClO–Wufeng systems, where pyrite/OM dissolution and associated micro-fracturing are key; additionally, links specific dissolution trends to measurable changes in compressive strength, elastic modulus, and fracability rather than focusing mainly on mineralogical changes.
This study	Uses NaClO (with comparison to H_2_O_2_ and KMnO_4_) to investigate oxidative dissolution of Wufeng shale cores from the Sichuan Basin; integrates gas permeability and porosity measurements, NMR-based pore-size distribution, contact-angle tests, and triaxial compression to evaluate changes in pore structure, wettability, and mechanical properties as a function of oxidant PV; additionally quantifies oxidant gel-breaking efficiency and develops a dynamic model of oxidation-enhanced imbibition and mechanical damage.	Provides a unified core-scale framework that simultaneously (i) links oxidative dissolution to the evolution of micro-to-meso-scale pore networks, (ii) quantifies the coupled response of wettability and mechanical strength, and (iii) incorporates oxidant gel-breaking behavior into a dynamic oxidation–imbibition model, thereby bridging the gap between pore-scale geochemical studies and purely mechanical or wettability studies and offering directly usable guidance for oxidation-assisted fracturing in Wufeng shale.

**Table 2:** Variation characteristics of shale wettability before and after displacement.

Core Number	Experimental Medium	Stabilization Time/min	Wetting Angle Before Dissolution/°	Wetting Angle After Dissolution/°	Degree of Hydrophilicity Increase/%	Wetting Type
1	Formation water	10.0	16.5	15.6	5.45	hydrophilic
2		10.0	19.2	17.2	10.42	hydrophilic
3		10.0	34.1	20.9	38.71	hydrophilic
4		10.0	33.3	16.2	51.35	hydrophilic
5		10.0	8.1	3.1	61.73	hydrophilic
6		10.0	25.1	12.9	48.61	hydrophilic

**Table 3:** Core NMR result data before displacement.

Core Number	Micropore T_2_ Spectrum Area	Medium Pore T_2_ Spectrum Area	Micropore Proportion/%	Medium Pore Proportion/%
0	25,626.17	923.78	96.52	3.48
1	26,679.99	836.80	96.96	3.04
2	26,857.71	1087.43	96.11	3.89
3	28,590.26	1249.09	95.81	4.19
4	26,972.72	1038.81	96.29	3.71
5	35,576.99	2032.95	94.59	5.41
6	24,896.12	945.92	96.34	3.66

**Table 4:** Core NMR result data after displacement.

Core Number	Micropore T_2_ Spectrum Area	Medium Pore T_2_ Spectrum Area	Micropore Proportion/%	Medium Pore Proportion/%
1	27,352.57	750.31	97.33	2.67
2	30,225.47	992.36	96.82	3.18
3	29,951.80	1236.60	96.04	3.96
4	30,301.01	1037.05	96.69	3.31
5	37,150.00	2368.37	94.01	5.99
6	27,728.69	967.12	96.63	3.37

**Table 5:** Changes in core pore proportion after displacement.

Core Number	Micropore Proportion Change/%	Medium Pore Proportion Change/%	Micropore Area Change/%	Medium Pore Area Change/%
1	0.37	−0.37	2.52	−10.33
2	0.71	−0.71	12.54	−8.74
3	0.23	−0.23	4.76	−1.00
4	0.40	−0.40	12.34	−0.17
5	−0.58	0.58	4.42	16.50
6	0.29	−0.29	11.38	2.24

**Table 6:** Triaxial compression results of rock.

Core Number	Displacement PV Number	Density/g/cm^3^	Confining Pressure/MPa	Compressive Strength/MPa	Elastic Modulus/GPa	Poisson’s Ratio
0	none	2.55	20	366.94	40.74	0.276
1	1 PV	2.55		363.68	39.92	0.274
2	2 PV	2.55		320.60	42.71	0.233
3	3 PV	2.55		351.70	42.98	0.288
4	4 PV	2.55		252.43	44.19	0.343
5	5 PV	2.52		154.85	28.33	0.215
6	6 PV	2.53		303.60	40.20	0.238

**Table 7:** Basic physical properties of core.

Core Number	Weight/g	Length/mm	Diameter/mm	Permeability/mD	Porosity/%
0	59.60	50.30	24.40	0.0004	2.83
1	59.53	50.24	24.42	0.0003	2.87
2	63.13	50.14	25.10	0.0003	3.21
3	58.94	50.04	24.42	0.0008	3.59
4	58.97	50.12	24.32	0.0009	3.35
5	61.82	49.34	25.20	0.0397	4.60
6	58.54	49.32	24.46	0.0003	3.13

**Table 8:** Change rate of shale quality after reaction of different oxidants.

Solution Type	Concentration /wt%	m_1_/g			m_2_/g			M/%		
		12 h	24 h	48 h	12 h	24 h	48 h	12 h	24 h	48 h
H_2_O_2_	10	2.003	2.004	2.010	1.973	1.961	1.980	1.51	2.13	1.50
	15	2.000	2.004	2.000	1.967	1.962	1.963	1.62	2.09	1.88
	20	2.000	2.000	2.000	1.963	1.967	1.963	1.83	1.69	1.85
KMnO_4_	1	2.000	2.001	2.003	2.052	2.089	2.130	−2.58	−4.39	−6.33
	2	2.000	2.003	2.004	2.086	2.154	2.203	−4.29	−7.55	−9.94
	4	2.001	2.001	2.003	2.157	2.245	2.309	−7.79	−12.17	−15.25
NaClO	6	2.002	2.001	2.000	1.954	1.942	1.939	2.42	2.92	3.04
	8	2.003	2.002	2.003	1.944	1.926	1.942	2.96	3.92	3.08
	10	2.001	2.001	2.003	1.959	1.931	1.943	2.11	3.53	3.00
Formation water	-	2.001	2.000	2.000	1.988	1.991	1.988	0.66	0.44	0.59

**Table 9:** Changes in ionic concentration in solution after reaction of different oxidants.

Solution Type	Concentration/wt%	Ca^2+^/mg/L			Mg^2+^/mg/L			Al^3+^/mg/L			SO_4_^2−^/mg/L		
		12 h	24 h	48 h	12 h	24 h	48 h	12 h	24 h	48 h	12 h	24 h	48 h
H_2_O_2_	10	260.0	292.2	281.1	10.8	12.5	12.5	0.1	0.3	0.1	619.1	672.1	740.6
	15	333.1	336.3	347.5	12.3	12.4	13.6	0.2	0.2	0.3	750.6	872.9	693.4
	20	397.0	422.5	387.0	13.2	13.9	13.7	0.4	0.1	0.2	942.9	961.4	770.0
KMnO_4_	1	2.0	1.1	3.2	0.3	0.2	0.3	0.0	0.0	0.1	440.1	642.4	788.1
	2	3.5	1.6	2.5	0.7	0.4	0.4	0.0	0.0	0.0	639.7	962.6	1063.1
	4	0.7	2.0	2.1	0.7	0.7	0.6	0.1	0.1	0.1	906.8	1141.0	1272.1
NaClO	6	13.1	9.5	10.5	1.2	0.9	0.9	0.0	0.0	0.0	1355.9	1389.5	1471.2
	8	10.7	7.6	8.4	1.4	0.8	0.7	0.1	0.0	0.0	1378.6	1293.4	1491.3
	10	8.3	7.9	7.2	1.1	0.9	0.7	0.0	0.0	0.0	1382.4	1595.1	1637.5
Formation water	-	41.1	42.4	52.1	3.7	4.3	6.7	0.1	0.5	0.1	92.2	99.7	134.3

## Data Availability

The raw experimental data underlying this study were generated under grants from the National Science and Technology Major Project (Grant No. 2025ZD1404305), the project of Theory of Hydrocarbon Enrichment under Multi-Spheric Interactions of the Earth (Grant No. THEMSIE04010107), and the Graduate Innovation Fund Project of Xi’an Shiyou University (Grant No. YCX2511001). Under the data-management and confidentiality provisions of the grant and the inter-institutional agreement, the primary datasets are temporarily restricted and cannot be publicly released until project completion and sponsor approval. Aggregated results, processed data, and methodological details sufficient to support the main findings are provided in the manuscript and Supplementary Materials.

## Supplementary Materials

The following supporting information can be downloaded at: https://www.mdpi.com/article/10.3390/gels11120982/s1, Figure S10. Core triaxial compression result curve; Table S2: Shale Wettability Test Graph Before and After Oxidative Dissolution; Table S3: Supplementary Raw NMR Data.
