# Supplementary figures and images for: Oxidative Dissolution Effects on Shale Pore Structure, Mechanical Properties, and Gel-Breaking Performance
Source: Gels. 2025 Dec 7;11(12):982. doi: 10.3390/gels11120982 (PMC12732822; doi:10.3390/gels11120982)

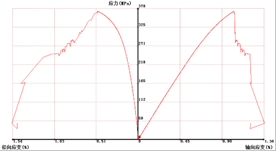

Supplement: Supplementary file 1 [file gels-11-00982-s001.zip › Figure S10. Core triaxial compression result curve/Core No. 0 Mechanical Test Results.png]

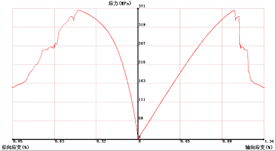

Supplement: Supplementary file 1 [file gels-11-00982-s001.zip › Figure S10. Core triaxial compression result curve/Core No. 1 Mechanical Test Results.png]

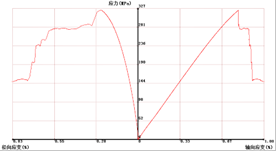

Supplement: Supplementary file 1 [file gels-11-00982-s001.zip › Figure S10. Core triaxial compression result curve/Core No. 2 Mechanical Test Results.png]

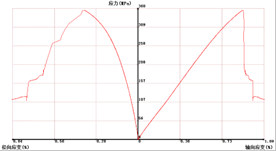

Supplement: Supplementary file 1 [file gels-11-00982-s001.zip › Figure S10. Core triaxial compression result curve/Core No. 3 Mechanical Test Results.png]

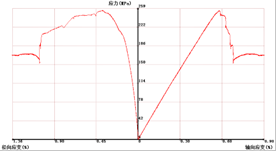

Supplement: Supplementary file 1 [file gels-11-00982-s001.zip › Figure S10. Core triaxial compression result curve/Core No. 4 Mechanical Test Results.png]

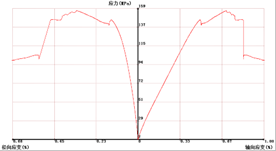

Supplement: Supplementary file 1 [file gels-11-00982-s001.zip › Figure S10. Core triaxial compression result curve/Core No. 5 Mechanical Test Results.png]

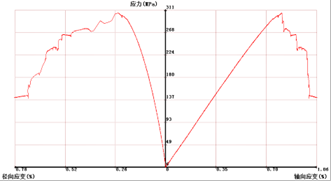

Supplement: Supplementary file 1 [file gels-11-00982-s001.zip › Figure S10. Core triaxial compression result curve/Core No. 6 Mechanical Test Results.png]

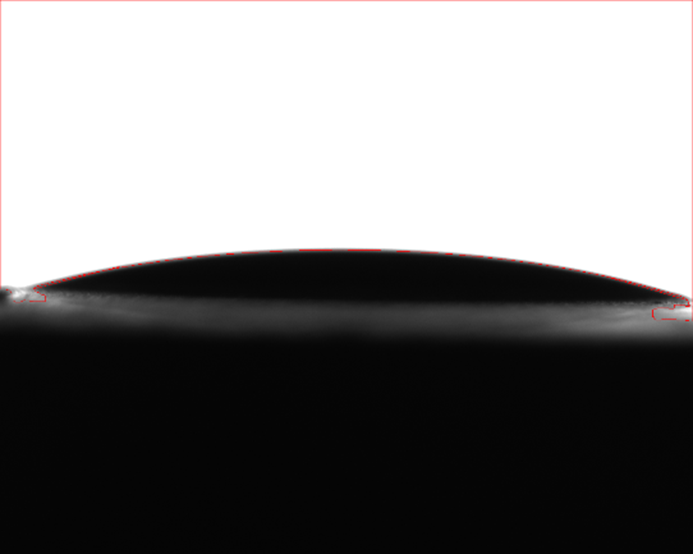

Supplement: Supplementary file 1 [file gels-11-00982-s001.zip › Table S2. Shale Wettability Test Graph Before and After Oxidative Dissolution/Core No. 1 Wettability Test Results Graph After Oxidative Dissolution.png]

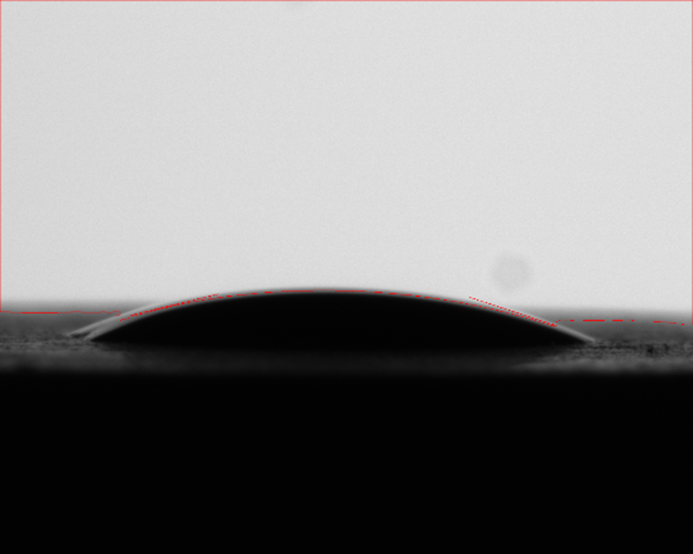

Supplement: Supplementary file 1 [file gels-11-00982-s001.zip › Table S2. Shale Wettability Test Graph Before and After Oxidative Dissolution/Core No. 1 Wettability Test Results Graph Before Oxidative Dissolution.png]

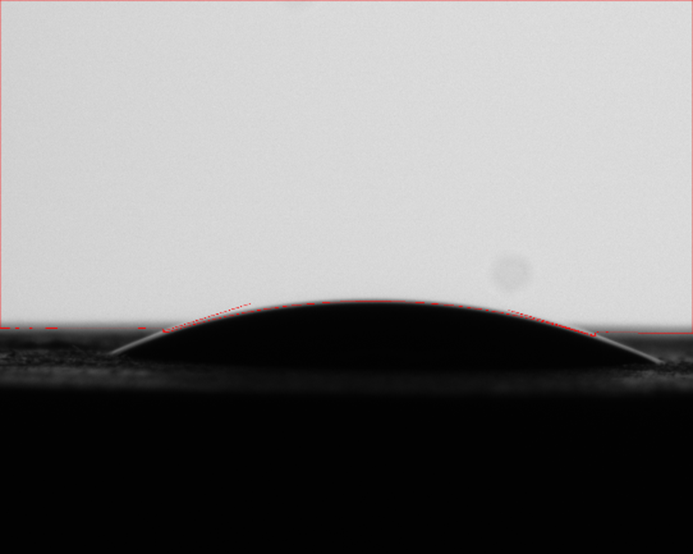

Supplement: Supplementary file 1 [file gels-11-00982-s001.zip › Table S2. Shale Wettability Test Graph Before and After Oxidative Dissolution/Core No. 2 Wettability Test Results Graph After Oxidative Dissolution.png]

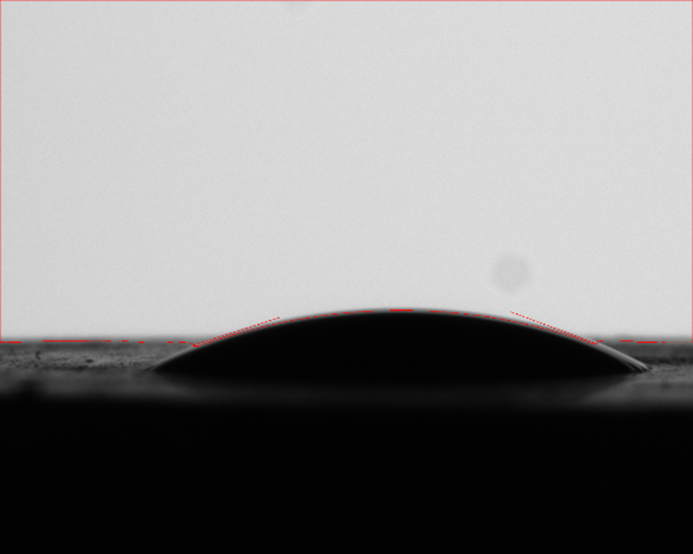

Supplement: Supplementary file 1 [file gels-11-00982-s001.zip › Table S2. Shale Wettability Test Graph Before and After Oxidative Dissolution/Core No. 2 Wettability Test Results Graph Before Oxidative Dissolution.png]

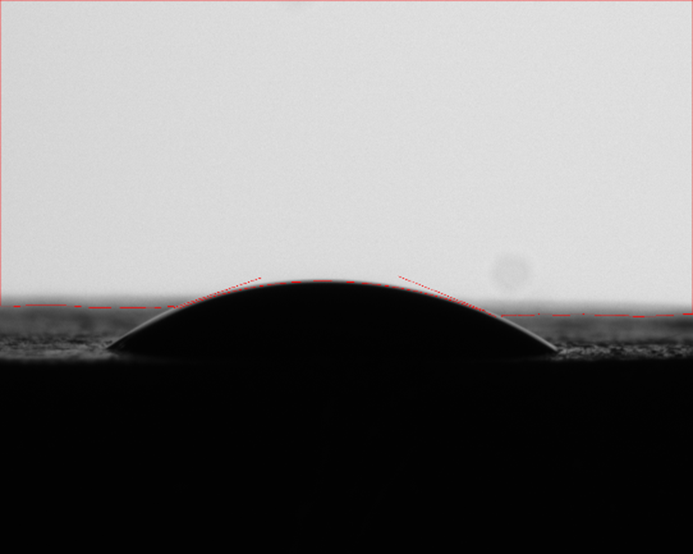

Supplement: Supplementary file 1 [file gels-11-00982-s001.zip › Table S2. Shale Wettability Test Graph Before and After Oxidative Dissolution/Core No. 3 Wettability Test Results Graph After Oxidative Dissolution.png]

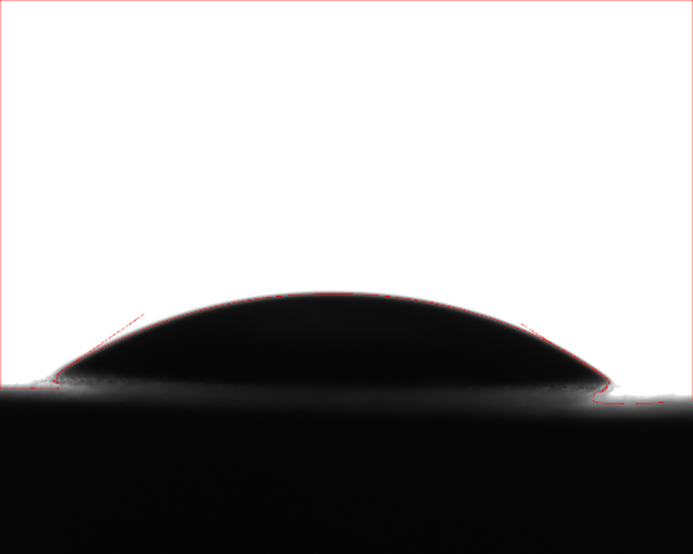

Supplement: Supplementary file 1 [file gels-11-00982-s001.zip › Table S2. Shale Wettability Test Graph Before and After Oxidative Dissolution/Core No. 3 Wettability Test Results Graph Before Oxidative Dissolution.png]

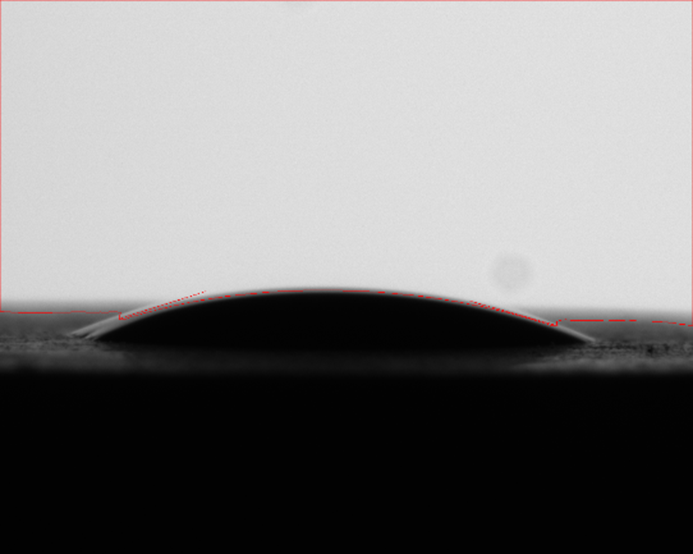

Supplement: Supplementary file 1 [file gels-11-00982-s001.zip › Table S2. Shale Wettability Test Graph Before and After Oxidative Dissolution/Core No. 4 Wettability Test Results Graph After Oxidative Dissolution.png]

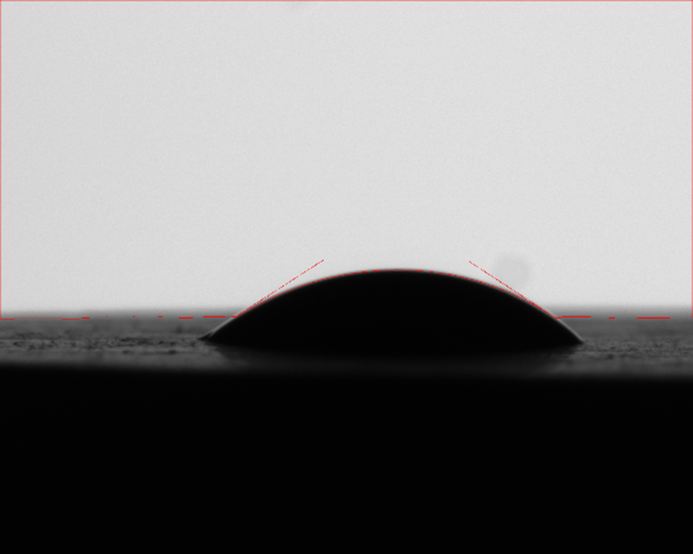

Supplement: Supplementary file 1 [file gels-11-00982-s001.zip › Table S2. Shale Wettability Test Graph Before and After Oxidative Dissolution/Core No. 4 Wettability Test Results Graph Before Oxidative Dissolution.png]

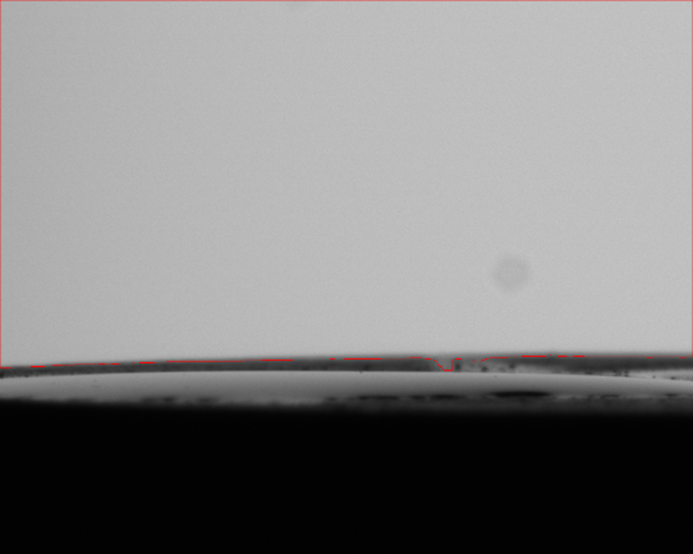

Supplement: Supplementary file 1 [file gels-11-00982-s001.zip › Table S2. Shale Wettability Test Graph Before and After Oxidative Dissolution/Core No. 5 Wettability Test Results Graph After Oxidative Dissolution.png]

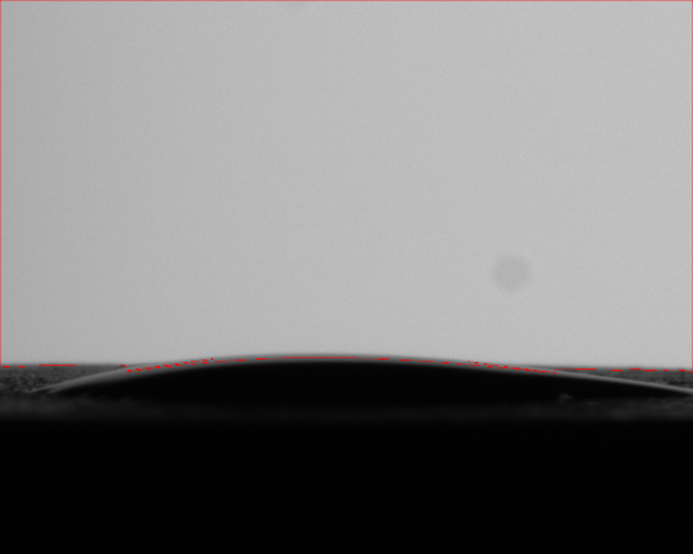

Supplement: Supplementary file 1 [file gels-11-00982-s001.zip › Table S2. Shale Wettability Test Graph Before and After Oxidative Dissolution/Core No. 5 Wettability Test Results Graph Before Oxidative Dissolution.png]

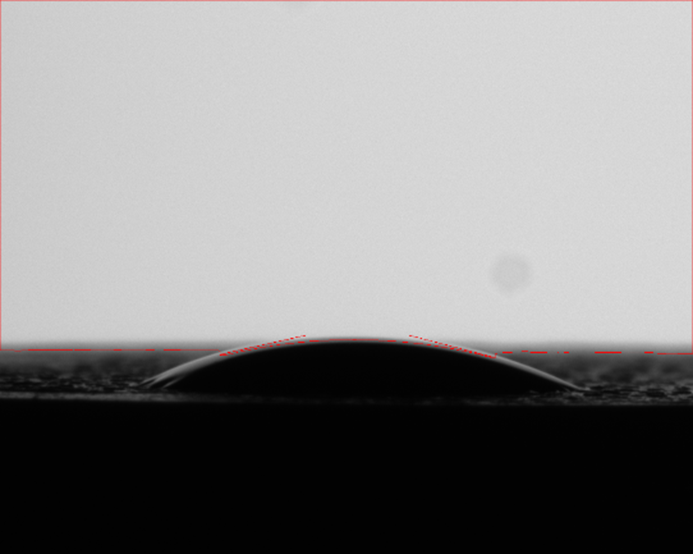

Supplement: Supplementary file 1 [file gels-11-00982-s001.zip › Table S2. Shale Wettability Test Graph Before and After Oxidative Dissolution/Core No. 6 Wettability Test Results Graph After Oxidative Dissolution.png]

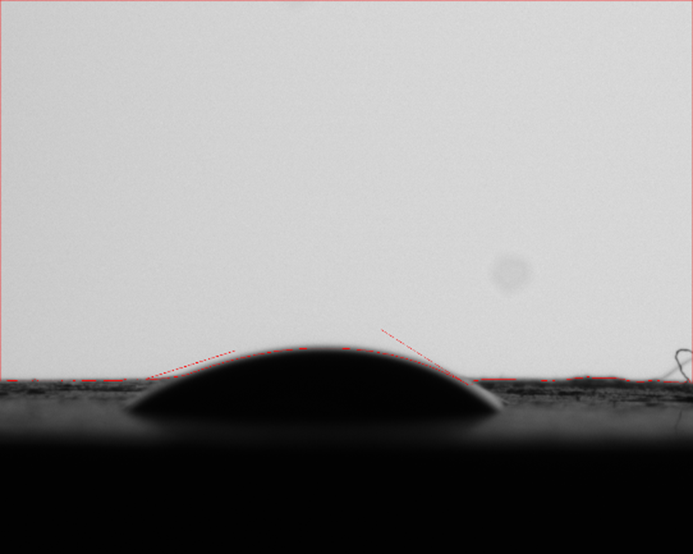

Supplement: Supplementary file 1 [file gels-11-00982-s001.zip › Table S2. Shale Wettability Test Graph Before and After Oxidative Dissolution/Core No. 6 Wettability Test Results Graph Before Oxidative Dissolution.png]
